# Comparison of methodological quality rating of systematic reviews on neuropathic pain using AMSTAR and R-AMSTAR

**DOI:** 10.1186/s12874-018-0493-y

**Published:** 2018-05-08

**Authors:** Svjetlana Dosenovic, Antonia Jelicic Kadic, Katarina Vucic, Nikolina Markovina, Dawid Pieper, Livia Puljak

**Affiliations:** 1 Department of Anesthesiology and Intensive Care Medicine, University Hospital Split, Split, Croatia; 2 Laboratory for Pain Research, University of Split School of Medicine, Soltanska 2, 21000 Split, Croatia; 3 Department of Pediatrics, University Hospital Split, Split, Croatia; 4 Agency for Medicinal Products and Medical Devices, Zagreb, Croatia; 5 Institute for Research in Operative Medicine (IFOM), Witten/Herdecke University, Cologne, Germany; 6 Agency for Quality and Accreditation in Health Care and Social Welfare, Zagreb, Croatia

**Keywords:** Neuropathic pain, Systematic review, Methodological quality, AMSTAR, R-AMSTAR, Interrater reliability

## Abstract

**Background:**

Systematic reviews (SRs) in the field of neuropathic pain (NeuP) are increasingly important for decision-making. However, methodological flaws in SRs can reduce the validity of conclusions. Hence, it is important to assess the methodological quality of NeuP SRs critically. Additionally, it remains unclear which assessment tool should be used. We studied the methodological quality of SRs published in the field of NeuP and compared two assessment tools.

**Methods:**

We systematically searched 5 electronic databases to identify SRs of randomized controlled trials of interventions for NeuP available up to March 2015. Two independent reviewers assessed the methodological quality of the studies using the Assessment of Multiple Systematic Reviews (AMSTAR) and the revised AMSTAR (R-AMSTAR) tools. The scores were converted to percentiles and ranked into 4 grades to allow comparison between the two checklists. Gwet’s AC1 coefficient was used for interrater reliability assessment.

**Results:**

The 97 included SRs had a wide range of methodological quality scores (AMSTAR median (IQR): 6 (5–8) vs. R-AMSTAR median (IQR): 30 (26–35)). The overall agreement score between the 2 raters was 0.62 (95% CI 0.39–0.86) for AMSTAR and 0.62 (95% CI 0.53–0.70) for R-AMSTAR. The 31 Cochrane systematic reviews (CSRs) were consistently ranked higher than the 66 non-Cochrane systematic reviews (NCSRs). The analysis of individual domains showed the best compliance in a comprehensive literature search (item 3) on both checklists. The results for the domain that was the least compliant differed: conflict of interest (item 11) was the item most poorly reported on AMSTAR vs. publication bias assessment (item 10) on R-AMSTAR. A high positive correlation between the total AMSTAR and R-AMSTAR scores for all SRs, as well as for CSRs and NCSRs, was observed.

**Conclusions:**

The methodological quality of analyzed SRs in the field of NeuP was not optimal, and CSRs had a higher quality than NCSRs. Both AMSTAR and R-AMSTAR tools produced comparable quality ratings. Our results point out to weaknesses in the methodology of existing SRs on interventions for the management NeuP and call for future improvement by better adherence to analyzed quality checklists, either AMSTAR or R-AMSTAR.

**Electronic supplementary material:**

The online version of this article (10.1186/s12874-018-0493-y) contains supplementary material, which is available to authorized users.

## Background

Systematic reviews (SRs) are considered to be of the highest quality in the hierarchy of evidence and are increasingly used for evidence-based decision making [1]. SRs should summarize the literature on a given topic using rigorous methodology. One of the standard features of SR methodology is the assessment of the quality of included primary studies, by using various tools [2].

However, there are also tools for assessing the methodological quality of SRs themselves, such as the Assessment of Multiple Systematic Reviews (AMSTAR), developed in 2007 [3]. AMSTAR was found to be a reliable and valid measurement tool for assessing the methodological quality of SRs [4]. A different group of authors suggested the use of revised AMSTAR (R-AMSTAR) in 2010 [5]. Despite the existence of R-AMSTAR, it was reported that AMSTAR had been used more frequently for the assessment of methodological quality of SRs [6]. This could be because AMSTAR, despite its flaws and many suggestions for its improvement, is shorter and simpler to use [7].

These tools for the assessment of methodological quality of SRs show that many SRs are inadequate. A comprehensive report on 300 SRs published in 2007 showed that their quality of reporting was inconsistent, indicating that readers should not think of SRs as synonymous with high-quality evidence [8]. An updated version of this study, published in 2016, indicated that the number of SRs being published is increasing, but the majority of them are still poorly conducted and reported [9].

Neuropathic pain (NeuP) has been estimated to affect 5–10% of the general population [10–12] and is associated with poor general health and quality of life [13–15]. This research area has received considerable attention from the International Association for the Study of Pain (IASP) as, despite the availability of many drugs and guidelines, NeuP remains under- or untreated in many cases [16]. Several evidence-based guidelines for the management of NeuP have been published in recent years [17–22]. It is of particular importance to ensure that those recommendations are based on high-quality research. It is also important to find out which measurement tool can be recommended for methodological quality rating in this cohort of interventional SRs.

Therefore, our primary aim was to analyze the methodological quality of SRs in the field of NeuP and to compare two different tools for quality assessment because it is still unclear which one is more appropriate. For this purpose, we used AMSTAR, a validated tool, and R-AMSTAR, which still cannot be put at the same level as AMSTAR with respect to validation. Our secondary aims were to calculate the interrater reliability and scoring discrepancies between the two authors and to analyze the overall agreement score between AMSTAR and R-AMSTAR. By assessing the methodological quality of available evidence, we hope to call attention to the current weaknesses of those tools and inspire future researchers to set higher standards in conducting SRs.

## Methods

### Protocol registration

We developed a protocol for this study a priori and registered it in the PROSPERO International Prospective Register of Systematic Reviews (registration number: CRD42015025832).

### Inclusion criteria

We included SRs of randomized controlled trials (RCTs) evaluating efficacy and safety of any therapeutic intervention for NeuP according to the IASP definition [23]. Any comparator and any outcome measure were eligible for inclusion. Studies in any language were eligible.

### Exclusion criteria

We excluded individual patient data SRs, SRs that were not interventional (e.g., prognostic, diagnostic accuracy), SRs that included other types of studies besides RCTs, as well as SRs that included a population with disorders that do not satisfy the current IASP criteria for NeuP [23]. We also excluded SRs published only as abstracts.

### Study selection

We searched MEDLINE, Cochrane Database of Systematic Reviews, DARE, CINAHL and PsycINFO from the earliest date that each database allowed up to March 9, 2015, without language or publication date restriction. A comprehensive search strategy for MEDLINE (Additional file 1) was developed by combining medical subject heading (MeSH) terms and text words for NeuP conditions with text words for SR/meta-analysis. The MEDLINE strategy was adapted for other databases. Two authors (SD, AJK) independently screened the search results for inclusion using pre-defined relevance criteria at all levels of screening (e.g., title and abstract, full-text review of potentially relevant articles). Discrepancies were resolved by discussion and the involvement of a third author (LP).

### Assessment of methodological quality

AMSTAR [24] and R-AMSTAR [5] were applied to all included SRs. We used a newer version of AMSTAR, with explanations available on the tool’s website [24]. Before quality assessment, the items on both scales were discussed, and a calibration exercise was performed on one of the included SRs. Summary scores for AMSTAR (possible range 0–11) and R-AMSTAR (possible range 11–44) were calculated by two independent junior research fellows (one clinician and one methodologist) experienced in methodological studies, but without formal experience with these tools (NM, KV). Raters without formal training in applying AMSTAR and R-AMSTAR were chosen because we did not want previous experience and potentially differing approaches to using AMSTAR and R-AMSTAR (due to ambivalence and multi-layer aspects of some domains) to influence the raters. In this way, the level of expertise of raters was removed as a potential confounding variable in rating the evidence. The AMSTAR tool was applied first to the whole set of included SRs. After approximately 4–6 weeks, the methodological quality rating was repeated with the R-AMSTAR tool. Discrepancies were resolved by the involvement of a third author (SD).

The performance of studies on each AMSTAR domain was rated by looking into the proportion of studies with a score “yes” compared to other possible scores, while the performance on each R-AMSTAR domain was assessed by looking into the percentage of studies with highest scores (scores 4 and 3), with 4 as the maximum score for a domain in R-AMSTAR. A subgroup analysis was made for the differences in methodological quality between Cochrane SRs (CSRs) and non-Cochrane SRs (NCSRs).

### Comparison of AMSTAR and R-AMSTAR

The AMSTAR and R-AMSTAR scores of each SR were subsequently converted to percentiles for each checklist to allow for the comparison of quality scores between the different assessment tools. We calculated percentiles by first ranking quality scores from lowest to highest and then taking the value from the ordered list that corresponds to that rank. The percentiles were assigned surrogate grades, as follows: grade A: ≥90%ile, grade B: 80–89%ile, grade C: 70–79%ile, grade D: ≤69%ile, as described previously [5]. Based on the resulting percentile scores, surrogate grades were assigned to each SR. Both total scores and surrogate grades were compared between the two assessment tools.

### Interrater agreement

We analyzed the interrater agreement by using Gwet’s AC1 coefficient [25]. An overall agreement score, as well as a score for each item, was calculated. We also calculated Cohen’s Kappa values and presented the results in Additional file 2 (AMSTAR) and Additional file 3 (R-AMSTAR). Values were calculated for AMSTAR by dichotomizing the responses into the categories “yes” (1 point) versus any other score (“no”, “can’t answer”, “not applicable”; each 0 points). For R-AMSTAR we calculated the interrater agreement on all the criteria in each of the 11 questions, resulting in a total of 41 assessments. The possible responses were “yes” or “no”. A study used for calibration exercise was excluded from the calculation [26].

For simplification, we interpreted both interrater agreement measures, Gwet’s AC1 coefficient and Cohen’s Kappa, as follows: coefficients of less than 0 signify poor agreement; 0.01–0.20 slight agreement; 0.21–0.40 fair agreement; 0.41–0.60 moderate agreement; 0.61–0.80 substantial agreement; and 0.81–0.99 almost perfect agreement, as proposed by Landis and Koch [27]. However, we need to emphasize that this categorization was originally proposed based on Cohen’s Kappa values only.

### Data analysis

The data about the performance of SRs on individual AMSTAR and R-AMSTAR domains were presented descriptively as frequencies and percentages. We presented methodological summary scores using median and interquartile range (IQR). Spearman rank correlation was performed to assess the association between total AMSTAR and R-AMSTAR scores.

We used IBM SPSS Statistics for Windows, version 19.0.0 (IBM Corp., Armonk, N.Y., USA) and R statistical software and available script files [28] for performing analyses. Statistical significance was defined at *P* < 0.05, two-tailed.

## Results

**Fig. 1:**
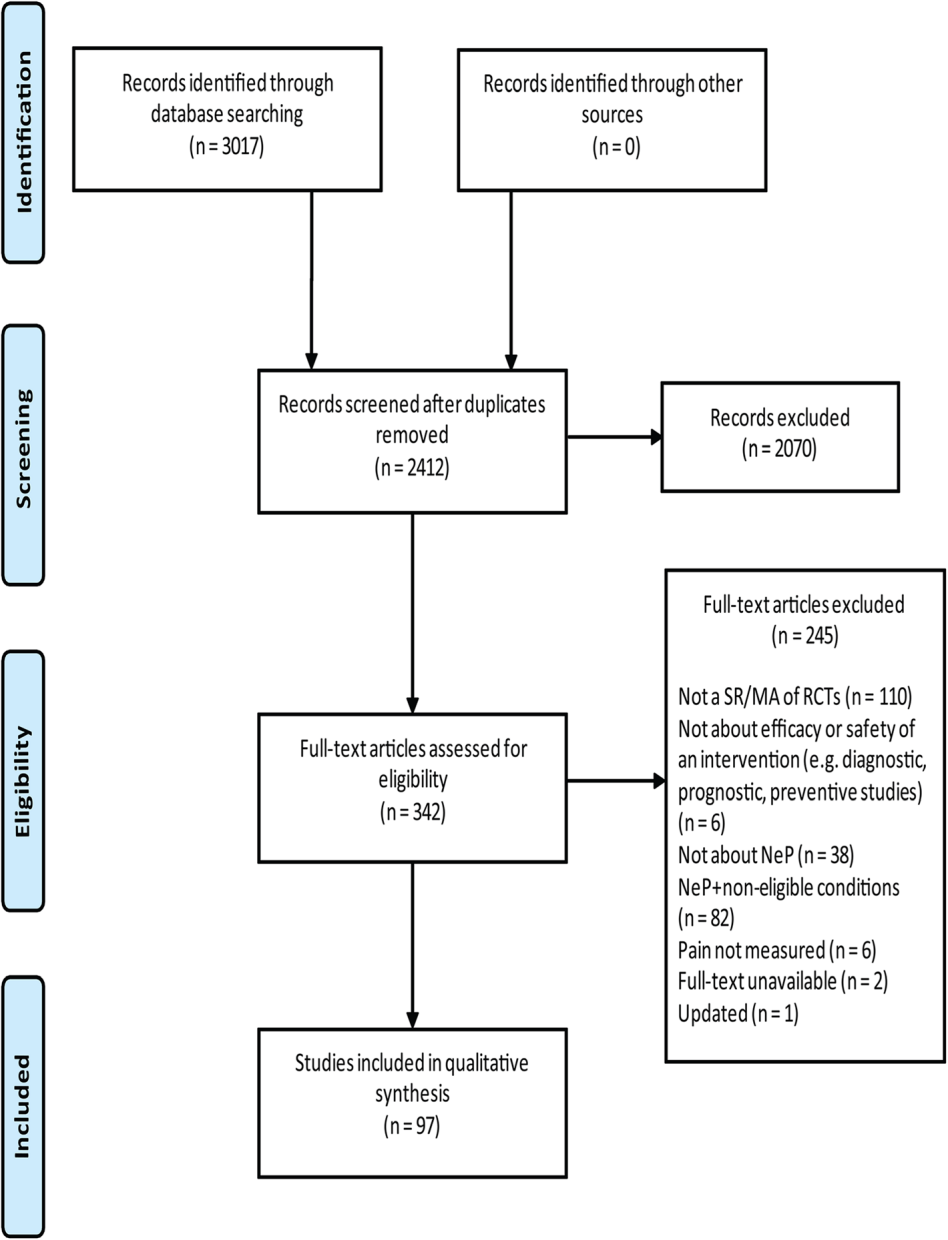
Study flow diagram

Ninety-seven SRs were included. Table 1 presents a summary description of the included SRs. The references of included studies are presented in Additional file 4, the list of excluded studies in Additional file 5, and the study selection process in Fig. 1.

**Table 1:** Summary characteristics of 97 included systematic reviews

Characteristic	N of SRs
Year	
1995–2000	4
2001–2005	10
2005–2010	27
2011–2015	56
Number of authors	
1	1
2–5	72
6–10	23
> 10	1
Language of the studies included in SRs	
Any language	49
English	30
English plus other	18
Not reported	6
Language of SRs	
English	91
Chinese	2
French	1
German	1
Spanish	1
Portuguese and English	1
Number of RCTs included	
0–1	8
2–10	37
11–20	30
21–30	14
31–40	4
41–100	3
> 100	1
Number of databases searched	
1	1
2–3	27
4–5	38
> 6	31
Neuropathic pain type	
Peripheral	86
Central	2
Peripheral and central	9
Total number of patients	
0	7
1–1000	28
1001–2000	28
2001–4000	14
> 4001	12
Unclear	8
Meta-analysis	
Yes	65
No	25
Not applicable (empty review)	7
Update of previous SR	
Yes	10
No	87
Number of updates	
1	7
2–3	2
> 4	1
Funding source	
Not reported	35
No external funding	13
Government	12
Foundation	12
None	11
Industry	8
Multiple	6

*SR* systematic review, *RCT* randomized controlled trial

### Methodological quality and adherence to individual AMSTAR and R-AMSTAR domains

The quality ratings of 97 included SRs are presented in Additional file 6 (AMSTAR) and Additional file 7 (R-AMSTAR). The median score was 6 (IQR: 5–8) on AMSTAR and 30 (IQR: 26–35) on R-AMSTAR.

A comparable quality rating was found based on surrogate grades assigned for AMSTAR and R-AMSTAR (Table 2). The lowest grade D was assigned to the majority of included NeuP SRs (64 based on AMSTAR vs. 68 based on R-AMSTAR assessment).

**Table 2:** Quality of all reviews, Cochrane and non-Cochrane systematic reviews according to AMSTAR and R-AMSTAR percentile scores

	All SRs		Cochrane SRs		Non-Cochrane SRs	
Grade	AMSTAR N (%)	R-AMSTAR N (%)	AMSTAR N (%)	R-AMSTAR N (%)	AMSTAR N (%)	R-AMSTAR N (%)
A	14 (14)	11 (11)	14 (45)	11 (36)	0 (0)	0 (0)
B	9 (9)	9 (9)	6 (19)	6 (19)	3 (5)	3 (5)
C	10 (10)	9 (9)	4 (13)	4 (13)	6 (9)	5 (8)
D	64 (66)	68 (70)	7 (23)	10 (32)	57 (86)	58 (88)
Total	97 (100)	97 (100)	31 (100)	31 (100)	66 (100)	66 (100)

Quality grades assigned according to percentiles (Grade A: ≥90%ile, Grade B: 80–89%ile, Grade C: 70–79%ile, Grade D: ≤ 69%ile)

*AMSTAR* Assessment of Multiple Systematic Reviews checklist, *R-AMSTAR* a revised version of AMSTAR checklist, *SR* systematic review

**Fig. 2:**
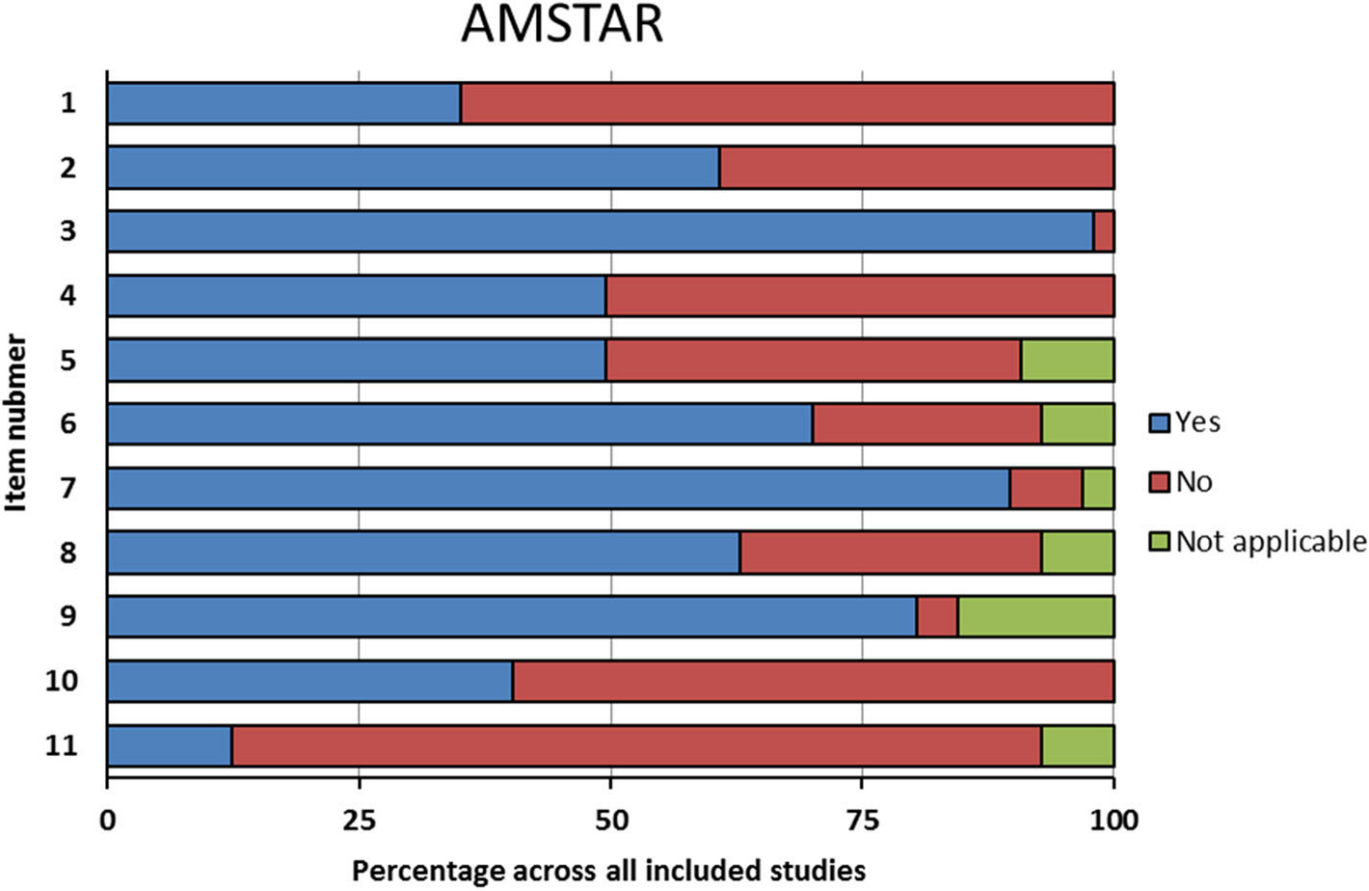
Quality of all included systematic reviews by AMSTAR domain

Studies scored best on AMSTAR items 3 (comprehensive literature search, 98% fulfilled), 7 (scientific quality assessed and documented, 89% fulfilled), and 9 (methods used to combine the findings appropriate, 80% fulfilled); and worst on items 11 (conflict of interest included, 12% fulfilled), 1 (‘a priori’ design provided, 35% fulfilled) and 10 (likelihood of publication bias assessed, 40% fulfilled) (Fig. 2).

**Fig. 3:**
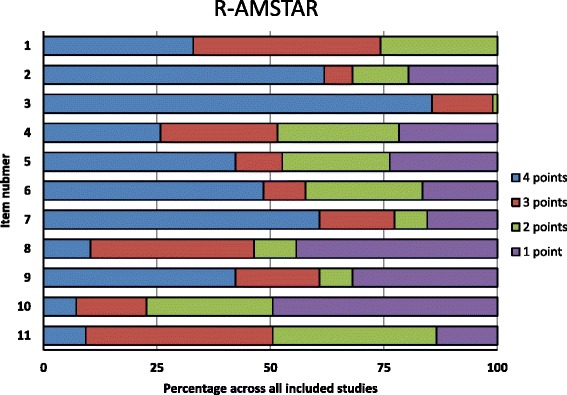
Quality of all included systematic reviews by R-AMSTAR domain

When R-AMSTAR was applied (Fig. 3), the best adherence was found for items 3 (comprehensive literature search, 86% of SRs with 4 points), and 2 (duplicate study selection and data extraction, 62% of SRs with 4 points); while the worst adherence was found for items 10 (likelihood of publication bias assessed, 49% of SRs with 1 point), and 8 (scientific quality of the included studies used appropriately in formulating conclusions, 44% of SRs with 1 point).

### Methodological quality of Cochrane systematic reviews (CSRs)

The median number of fulfilled items in 31 CSRs was 9 (IQR: 8–10) of 11 on AMSTAR and 37 (IQR: 33–40) of 44 maximum possible R-AMSTAR items.

When surrogate grades were assigned, the majority of CSRs were rated as grade A on AMSTAR (*N* = 14) and R-AMSTAR (*N* = 11). The distribution of other grades was also similar (Table 2).

**Fig. 4:**
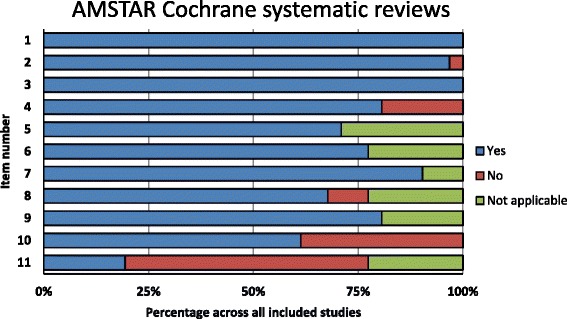
Quality of Cochrane systematic reviews by AMSTAR domain

**Fig. 5:**
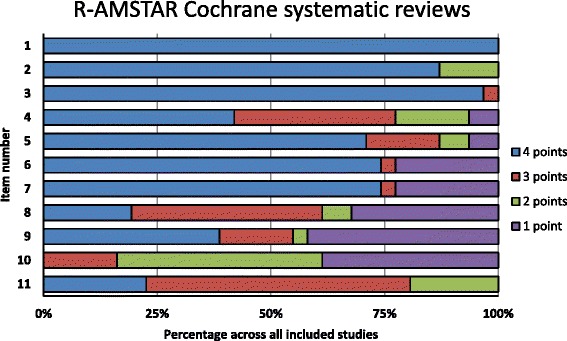
Quality of Cochrane systematic reviews by R-AMSTAR domain

All CSRs scored “yes” on AMSTAR items 1 (‘a priori’ design provided) and 3 (comprehensive literature search), and 30 on item 2 (duplicate study selection and data extraction) (Fig. 4). Similar results were found for R-AMSTAR: all CSRs scored 4 points on item 1, 30 on item 3, and 27 on item 2 (Fig. 5).

The worst compliance for CSRs was on AMSTAR item 11, with 18 studies that did not include conflict of interest in both the SR and the included studies (Fig. 4). On R-AMSTAR (Fig. 5), the domains with the poorest performance were domains 9 (appropriate methods used to combine the findings of studies), 10 (likelihood of publication bias assessed), and 8 (scientific quality appropriately used in formulating conclusions).

### Methodological quality of non-Cochrane systematic reviews (NCSRs)

The 66 NCSRs fulfilled a median of 6 (IQR: 5–7) of 11 possible items on AMSTAR and 29 (IQR: 25–32) of maximum 44 items on R-AMSTAR.

As shown in Table 2, none of the NCSRs reached grade A, while the majority received grade D on both AMSTAR (*N* = 57) and R-AMSTAR (*N* = 58). Grading based on the AMSTAR and R-AMSTAR scores yielded almost identical numbers of grade B and C NCSRs.

**Fig. 6:**
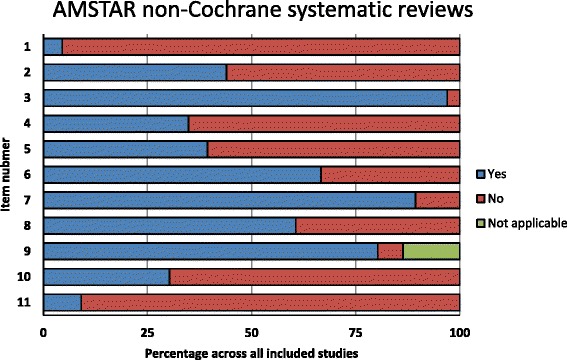
Quality of non-Cochrane systematic reviews by AMSTAR domain

**Fig. 7:**
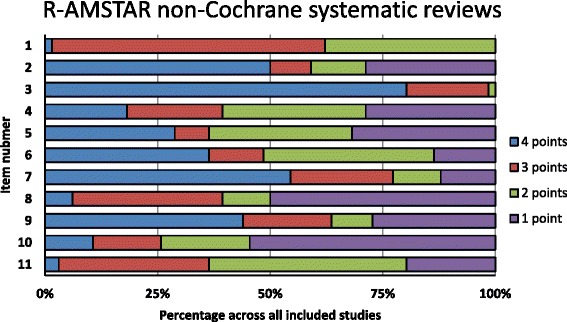
Quality of non-Cochrane systematic reviews by R-AMSTAR domain

The NCSRs showed the best compliance with items 3 (comprehensive literature search), 7 (scientific quality of the included studies assessed and documented), and 9 (methods used to combine the findings of studies appropriate) on both AMSTAR (Fig. 6) and R-AMSTAR (Fig. 7). The poorest performing domains on AMSTAR were items 1 (‘a priori’ design provided) and 11 (conflict of interest included), with more than 90% of studies that did not fulfill criteria for a “yes” score (Fig. 6). Figure 7 shows the poorest performing domains on R-AMSTAR: items 10 (likelihood of publication bias assessed) and 8 (scientific quality of the included studies used appropriately in formulating conclusions).

### Comparison of methodological quality of Cochrane and non-Cochrane systematic reviews

The quality ratings of 97 SRs expressed in percentiles were similar between AMSTAR (median 47.94, IQR 27.84–71.13) and R-AMSTAR (median 50, IQR 26.3–74.2). Cochrane SRs consistently scored higher than NCSRs, and similar ratings were obtained using both AMSTAR (CSRs median (IQR): 80.93 (71.13–90.72) vs. NCSRs median (IQR): 47.94 (27.84–61.86)) and R-AMSTAR (CSRs median (IQR): 83 (61.3–92.15) vs. NCSRs median (IQR): 42.3 (20.1–57.7)).

### Correlation of AMSTAR and R-AMSTAR ratings

We found significant high positive correlation between the AMSTAR and R-AMSTAR scores for all analyzed SRs (Spearman’s rho = 0.88, 95% CI 0.83–0.92; *P* < 0.001), as well as for CSRs (Spearman’s rho = 0.82, 95% CI 0.65–0.91; *P* < 0.001) and NCSRs (Spearman’s rho = 0.75, 95% CI 0.61–0.84; *P* < 0.001).

### Interrater agreement

The levels of agreement between the 2 raters on both tools ranged from poor to almost perfect. The overall agreement score was substantial for both AMSTAR (Gwet’s AC1 = 0.62, 95%CI 0.39–0.86) and R-AMSTAR (Gwet’s AC1 = 0.62, 95%CI 0.53–0.70). Detailed interrater agreement scores for all items on AMSTAR and R-AMSTAR are presented in Tables 3 and 4, respectively.

**Table 3:** Interrater agreement for AMSTAR

Item	Gwet’s AC1	SEM	95% CI
1. Was an ‘a priori’ design provided	0.90	0.04	0.82–0.99
2. Was there duplicate study selection and data extraction	0.47	0.09	0.29–0.66
3. Was a comprehensive literature search performed	0.92	0.03	0.85–0.98
4. Was the status of publication (i.e. grey literature) used as an inclusion criterion	0.03	0.11	−0.19-0.24
5. Was a list of studies (included and excluded) provided	0.63	0.08	0.47–0.79
6. Were the characteristics of the included studies provided	−0.09	0.10	−0.30-0.12
7. Was the scientific quality of the included studies assessed and documented	0.89	0.04	0.81–0.97
8. Was the scientific quality of the included studies used appropriately in formulating conclusions	0.68	0.08	0.53–0.83
9. Were the methods used to combine the findings of studies appropriate	0.88	0.04	0.79–0.96
10. Was the likelihood of publication bias assessed	0.76	0.07	0.62–0.89
11. Was the conflict of interest included	0.80	0.05	0.70–0.91
Overall agreement (mean score of 11 items)	0.62	0.10	0.39–0.86

*AMSTAR* Assessment of Multiple Systematic Reviews checklist, *SEM* standard error of the mean, *CI* confidence interval

**Table 4:** Interrater agreement for R-AMSTAR

Item	Criterion	Gwet’s AC1	SEM	95% CI
1. Was an ‘a priori’ design provided	A	0.02	0.11	−0.20-0.24
	B	0.93	0.03	0.88–0.99
	C	0.34	0.10	0.15–0.54
2. Was there duplicate study selection and data extraction	A	0.78	0.06	0.66–0.90
	B	0.64	0.08	0.48–0.79
	C	0.62	0.08	0.45–0.78
3. Was a comprehensive literature search performed	A	0.97	0.02	0.93–1.00
	B	0.94	0.03	0.89–0.99
	C	0.25	0.11	0.03–0.47
	D	0.55	0.09	0.38–0.73
	E	0.83	0.05	0.73–0.93
4. Was the status of publication (i.e. grey literature) used as an inclusion criterion	A	0.55	0.09	0.38–0.72
	B	−0.32	0.10	−0.52-(−0.12)
	C	0.24	0.10	0.04–0.44
	D	0.75	0.07	0.61–0.88
5. Was a list of studies (included and excluded) provided	A	0.95	0.02	0.89–1
	B	0.79	0.06	0.67–0.92
	C	0.41	0.10	0.21–0.60
	D	0.48	0.09	0.30–0.66
6. Were the characteristics of the included studies provided	A	0.79	0.06	0.68–0.91
	B	0.46	0.09	0.28–0.64
	C	0.60	0.08	0.43–0.76
7. Was the scientific quality of the included studies assessed and documented	A	0.86	0.04	0.77–0.95
	B	0.66	0.08	0.50–0.81
	C	0.61	0.08	0.45–0.78
	D	0.73	0.07	0.59–0.86
8. Was the scientific quality of the included studies used appropriately in formulating conclusions	A	0.66	0.08	0.50–0.81
	B	0.23	0.10	0.03–0.43
	C	0.28	0.10	0.07–0.48
	D	0.97	0.02	0.93–1
9. Were the methods used to combine the findings of studies appropriate	A	0.71	0.07	0.57–0.85
	B	0.74	0.07	0.60–0.88
	C	0.68	0.08	0.53–0.83
	D	0.67	0.08	0.52–0.82
	E	0.47	0.10	0.28–0.66
10. Was the likelihood of publication bias assessed	A	0.58	0.08	0.41–0.74
	B	0.80	0.06	0.69–0.92
	C	0.95	0.03	0.90–1
11. Was the conflict of interest included	A	0.70	0.07	0.56–0.84
	B	0.60	0.08	0.44–0.76
	C	0.85	0.05	0.76–0.95
Overall agreement (mean score of 41 items)		0.62	0.04	0.53–0.70

*R-AMSTAR*, a revised version of Assessment of Multiple Systematic Reviews checklist, *SEM* standard error of the mean, *CI* confidence interval

Four AMSTAR domains reached almost perfect Gwet’s AC1 values: ‘a priori’ design of the research question (item 1), comprehensive literature search (item 3), assessment of the scientific quality of the included studies (item 7), and the use of appropriate methods for combining the findings of studies (item 9). On the other hand, poor agreement was observed when raters judged whether the status of publication was used as an inclusion criterion (item 4) and if the characteristics of the included studies were provided (item 6).

Based on Gwet’s AC1 values, items 3, 7, 10 (publication bias assessment) and 11 (information about conflict of interest) had the highest average Gwet’s AC1 coefficients (≥0.7), and item 4 the lowest (0.31) on R-AMSTAR.

The assessment of the fulfillment of individual criteria within each R-AMSTAR item showed that poor agreement was achieved for criteria 4B (statement of exclusion of any reports based on the publication status, language, etc.) and 1A (‘a priori’ design provided).

Almost perfect agreement (Gwet’s AC1 0.81–1.00) was observed for criteria 1B (statement of inclusion criteria), 3A (at least two electronic sources searched); 3B (reporting years and databases searched), 3E (manual journal search), 5A (Table/list/or figure of included studies provided), 7A (‘a priori’ methods of assessment of scientific quality provided), 8D (whether clinical consensus statement drives toward revision or confirmation of clinical practice guidelines), 10 C (statistical tests for publication bias assessment), and 11C (conflict of interest assessed in included studies).

## Discussion

We found that the methodological quality of analyzed SRs published in the field of NeuP was not optimal. When we compared AMSTAR and R-AMSTAR, we found that, overall, both tools produced comparable quality ratings of the included NeuP SRs.

Several previous studies that focused only on the methodological quality of SRs in other fields of pain used AMSTAR. For example, Martinis et al. assessed the quality of 40 SRs about surgical treatment of low back pain. They reported that 5% of SRs were of excellent quality, most were of fair quality, and 22.5% of poor quality [29]. Song et al. analyzed the methodological quality of 17 SRs on the effectiveness of non-pharmacological cancer pain management and found that only 1 SR was of high quality, while five were of low quality. The mean AMSTAR score was 5.47, indicating overall moderate quality [30]. We could not find any studies that focused only on methodological quality in the field of pain that used R-AMSTAR, and none that compared the use of the 2 measurement tools.

A recent SR of Pieper et al. found that AMSTAR had good measurement properties, but R-AMSTAR did not [31]. Pieper et al. searched four databases to analyze reliability, validity, and feasibility of the AMSTAR and R-AMSTAR. They included 9 studies that analyzed AMSTAR, two studies that analyzed R-AMSTAR, and one article that analyzed both tools. The authors of that SR did not provide any methodological details about calculating interrater reliability for R-AMSTAR because the studies using R-AMSTAR did not report them either [31].

Without any guidance for calculating interrater reliability in the R-AMSTAR, and led by the findings from a study by Wongpakaran et al. that showed Gwet’s AC1 coefficient was a more stable interrater reliability measure than Cohen’s Kappa in personality disorder samples [32], we decided to use Gwet’s AC1 in our study. However, we have to take into account that the interpretation of reliability measures by Landis and Koch was originally published for measures of Cohen’s Kappa and it might not be fully applicable for Gwet’s AC1 coefficients. Compared to Cohen’s Kappa, Gwet’s AC1 values tend to be higher [32].

Our results showed poor agreement for AMSTAR item 4 (status of publication was used as an inclusion criterion) and 6 (characteristics of the included studies). Low interrater reliability using Cohen’s Kappa coefficient has been previously reported for item 4, and the study mentioned difficulties in applying the publication status item [4].

Findings from a 2015 SR by Pieper et al., based on results from 6 studies, also showed the lowest median interrater reliability using Cohen’s Kappa for AMSTAR item 6 (characteristics of the included studies) [31]. Other items with lowest median interrater reliability scores (indicating substantial agreement) were item 8 (scientific quality appropriately used in formulating conclusions) and 5 (list of included and excluded studies), which is in accordance with our interrater reliability measures. The highest interrater reliability scores (almost perfect agreement) in the study of Pieper et al. were reported for item 10 (publication bias assessment) and 11 (conflicts of interest stated); those items reached substantial agreement on our sample of 97 SRs [31].

The raters reached substantial agreement on 4 of our R-AMSTAR items with the highest average Gwet’s AC1 values (items 3, 7, 10, 11). Compared to Cohen’s Kappa values obtained in an SR in subfertility [33], only item 10 in their study had a higher interrater reliability.

For R-AMSTAR we calculated the interrater agreement on 41 items by taking into account all the criteria (from 3 (A-C) to 5 criteria (A-E)) in each of the 11 items. We cannot correctly judge whether the raters agree that the same criteria are fulfilled (e.g., rater 1: A and B; rater 2: C and D) solely by looking into the 11-item based agreement. Our results indicate the most problematic individual criteria within each R-AMSTAR item where our raters obtained poor agreement; they are the statement of exclusion of any reports based on the publication status, language, etc. (criterion 4B) and ‘a priori’ design provided (criterion 1A). These items would particularly benefit from further clarification on how to apply them.

We also confirmed in our sample of NeuP interventional SRs that CSRs have a higher methodological quality compared to NCSRs: the majority of CSRs had scores above the 80th percentile, compared to NCSRs where the majority scored in the lowest category. A number of studies from different research fields have previously shown that the quality of CSRs is superior [4, 8, 34–36]. However, we did not find a difference in correlation measures between AMSTAR and R-AMSTAR depending on whether it is a Cochrane review or not. In the study of Popovich et al., a much higher correlation between AMSTAR and R-AMSTAR was found for NCSRs than for CSRs in the field of subfertility. These results warrant further studying [31].

In order to improve the methodological quality of SRs, a joint action by SR authors, peer reviewers, and editors is needed. When planning and reporting their SR, the authors should follow a relevant checklist to ensure that the manuscript has appropriate methodological quality. Peer reviewers should analyze SRs against the relevant checklist, and editors could endorse methodological quality tools for specific article types and require authors to adhere to them. Editors are gatekeepers who can make sure the authors adhere to expected submission criteria. A recent editorial described that one journal is beginning this process: inspired by the findings of a study that showed poor and stagnating methodological quality of SRs in the field of urology [37], the BJU International decided to request from all SR authors to submit an AMSTAR checklist together with the manuscript, which will be utilized as part of the editorial and peer-review process [38].

A limitation of this study is that we have included SRs published since 1995, while AMSTAR was only published in 2007. It is possible that the methodological quality of SRs has increased since the introduction of AMSTAR since there is already some evidence that might support this [39–42]. Another limitation of this study is that calculating AMSTAR scores in this way implies that each item has the same weight. This assumption might be wrong because not all the domains of AMSTAR should have the same methodological value.

It is also possible that the differences observed between interrater agreement results obtained in ours and other studies might be influenced by the experience of the raters [43], although this is difficult to judge since rater experience is not frequently reported in the included studies [31]. We believe that both tools could be easily applied by an untrained person after a thorough discussion of the items and a calibration exercise before assessment, as we did.

Future studies should also include an assessment of ROBIS, a new tool for assessing the risk of bias in SRs, which was published in 2016 [44]. Likewise, the development of AMSTAR 2 was announced at the 2016 Cochrane Colloquium [45], so new studies about the updated tool are warranted once it is published.

## Conclusion

In conclusion, since both AMSTAR and R-AMSTAR tools produced comparable quality ratings of the included SRs, we advise future use of AMSTAR because it is shorter and it has been shown to have good measurement properties in previous studies, unlike R-AMSTAR. Our analysis indicated that the methodological quality of existing SRs about interventions for the management NeuP is suboptimal, which calls for improvement by better adherence to methodological quality criteria.

## Additional files

Additional file 1: Search strategy for MEDLINE. (DOCX 23 kb)

Additional file 2: Interrater agreement (Cohen’s kappa) for AMSTAR. (DOCX 22 kb)

Additional file 3: Interrater agreement (Cohen’s kappa) for R-AMSTAR. (DOCX 28 kb)

Additional file 4: References of included studies. (DOCX 139 kb)

Additional file 5: List of excluded studies with reasons. (DOCX 296 kb)

Additional file 6: AMSTAR rating for each study. (DOCX 211 kb)

Additional file 7: R-AMSTAR rating for each study. (DOCX 221 kb)

## Abbreviations

AC1: Gwet’s AC1 interrater agreement coefficient
AMSTAR: The Assessment of Multiple Systematic Reviews
CSR: Cochrane systematic review
IASP: International Association for the Study of Pain
IQR: Interquartile range
MeSH: Medical Subject Headings
NCSR: Non-Cochrane systematic review
NeuP: Neuropathic pain
PROSPERO: International prospective register of systematic reviews
R-AMSTAR: Revised AMSTAR
RCT: Randomized controlled trial
ROBIS: Tool to assess risk of bias in systematic reviews
SR: Systematic review
