# Supplementary material for: Comparison of methodological quality rating of systematic reviews on neuropathic pain using AMSTAR and R-AMSTAR
Source: BMC Med Res Methodol. 2018 May 8;18:37. doi: 10.1186/s12874-018-0493-y (PMC5941595; doi:10.1186/s12874-018-0493-y)
Supplement: Supplementary file 1 — Search strategy for MEDLINE. (DOCX 23 kb) [file 12874_2018_493_MOESM1_ESM.docx]

**Additional file 1**. Search strategy for MEDLINE.

Ovid MEDLINE(R) In-Process & Other Non-Indexed Citations and Ovid MEDLINE(R) 1946 to Present (27.02.2015.)

1 exp Neuralgia/

2 Neuropathic pain$.mp

3 (neuropath$3 adj5 pain$).mp.

4 Neuropath$3.mp.

5 neuralg$.mp.

6 (neurogen$ adj3 pain$).mp.

7 Neurodyni$.mp.

8 Nerve pain.mp

9 pain nerve.mp.

10 Diabetic Neuropathies/

11 (diabet$ adj3 neuropath$3).mp.

12 (postherp$ adj3 neuralg$).mp.

13 (trigemin$ adj3 neuralg$).mp.

14 ((facial$ or face) adj3 (pain$ or neuralg$)).mp.

15 Burning Mouth Syndrome/

16 (burning adj3 mouth$).mp.

17 (HIV adj3 neuropath$3).mp.

18 (neuropath$3 adj3 cancer$ adj3 pain$).mp.

19 (pain$ adj3 neuropath$3 adj3 (post-treatment$ or post treatment$ or posttreatment$ or surg$ or post-op$ or postop$ or post op$)).mp.

20 Phantom limb/

21 (phantom adj3 limb$).mp.

22 Polyneuropathies/

23 (pain$ adj3 polyneuropath$3).mp.

24 exp Nerve Compression Syndromes/

25 exp Peripheral Nervous System Diseases/

26 ((compress$ or peripher$) adj3 (Neuropath$3 or nerv$)).mp.

27 Spinal Cord Injuries/

28 (spinal cord adj3 (injury or injuries or injured)).mp.

29 ((post amputation or post-amputation or postamputation) adj3 pain$).mp.

30 (stroke$ adj3 pain$).mp.

31 (idiopathic$ adj3 (pain$ or Neuropath$3)).mp.

32 exp Multiple Sclerosis/

33 multiple sclerosis.mp.

34 Stroke/

35 Radiculopathy/

36 (radiculopath$ or radicular pain$).mp.

37 exp Complex regional pain syndromes/

38 (complex adj3 region$ adj3 pain$).mp.

39 CRPS.mp.

40 (hand$ adj3 shoulder$ adj3 syndrom$).mp.

41 causalgi$.mp.

42 pain$.mp.

43 (4 or 10 or 15 or 16 or 20 or 22 or 24 or 25 or 27 or 28 or 32 or 33 or 34 or 40) and 42

44 1 or 2 or 3 or 5 or 6 or 7 or 8 or 9 or 11 or 12 or 13 or 14 or 17 or 18 or 19 or 21 or 23 or 26 or 29 or 30 or 31 or 35 or 36 or 37 or 38 or 39 or 41

45 43 or 44

46 Hyperalgesi$.mp.

47 allodynia$.mp.

48 46 or 47

49 45 or 48

50 (review or review,tutorial or review, academic).pt.

51 (medline or medlars or embase or pubmed or cochrane).tw,sh.

52 (scisearch or psychinfo or psycinfo).tw,sh.

53 (psychlit or psyclit).tw,sh.

54 cinahl.tw,sh.

55 ((hand adj2 search$) or (manual$ adj2 search$)).tw,sh.

56 (electronic database$ or bibliographic database$ or computeri?ed database$ or online database$).tw,sh.

57 (pooling or pooled or mantel haenszel).tw,sh.

58 (peto or dersimonian or der simonian or fixed effect).tw,sh.

59 (retraction of publication or retracted publication).pt.

60 or/51-59

61 50 and 60

62 meta-analysis.pt.

63 meta-analysis.sh.

64 (meta-analys$ or meta analys$ or metaanalys$).tw,sh.

65 (systematic$ adj5 review$).tw,sh.

66 (systematic$ adj5 overview$).tw,sh.

67 (quantitativ$ adj5 review$).tw,sh.

68 (quantitativ$ adj5 overview$).tw,sh.

69 (quantitativ$ adj5 synthesis$).tw,sh.

70 (methodologic$ adj5 review$).tw,sh.

71 (methodologic$ adj5 overview$).tw,sh.

72 (integrative research review$ or research integration).tw.

73 or/62-72

74 61 or 73

75 Comment/

76 Letter/

77 Editorial/

78 Guideline/

79 or/75-78

80 74 not 79

81 49 and 80

82 remove duplicates from 81

**Abbreviations:** HIV: Human Immunodeficiency Virus; CRPS: complex regional pain syndrome.
