# Supplementary material for: Comparison of methodological quality rating of systematic reviews on neuropathic pain using AMSTAR and R-AMSTAR
Source: BMC Med Res Methodol. 2018 May 8;18:37. doi: 10.1186/s12874-018-0493-y (PMC5941595; doi:10.1186/s12874-018-0493-y)
Supplement: Supplementary file 2 — Interrater agreement (Cohen’s kappa) for AMSTAR. (DOCX 22 kb) [file 12874_2018_493_MOESM2_ESM.docx]

**Additional file 2.** Interrater agreement (Cohen’s kappa) for AMSTAR.

| **Item** | **Kappa** | **SEM** | **95% CI** |
| --- | --- | --- | --- |
| 1. Was an 'a priori' design provided | 0.89 | 0.05 | 0.79-0.98 |
| 2. Was there duplicate study selection and data extraction | 0.42 | 0.09 | 0.24-0.59 |
| 3. Was a comprehensive literature search performed | 0.33 | 0.19 | -0.05-0.70 |
| 4. Was the status of publication (i.e. grey literature) used as an inclusion criterion | 0.15 | 0.06 | 0.04-0.27 |
| 5. Was a list of studies (included and excluded) provided | 0.62 | 0.08 | 0.46-0.78 |
| 6. Were the characteristics of the included studies provided | 0.15 | 0.04 | 0.06-0.23 |
| 7. Was the scientific quality of the included studies assessed and documented | 0.65 | 0.11 | 0.42-0.87 |
| 8. Was the scientific quality of the included studies used appropriately in formulating conclusions | 0.61 | 0.09 | 0.44-0.77 |
| 9. Were the methods used to combine the findings of studies appropriate | 0.75 | 0.08 | 0.59-0.91 |
| 10. Was the likelihood of publication bias assessed | 0.75 | 0.07 | 0.61-0.88 |
| 11. Was the conflict of interest included | 0.26 | 0.14 | -0.01-0.53 |
| Overall agreement (mean score of 11 items) | 0.51 | 0.08 | 0.33 -0.68 |

**Abbreviations:** AMSTAR, Assessment of Multiple Systematic Reviews checklist; SEM, standard error of the mean; CI, confidence interval.
