# Supplementary material for: Comparison of methodological quality rating of systematic reviews on neuropathic pain using AMSTAR and R-AMSTAR
Source: BMC Med Res Methodol. 2018 May 8;18:37. doi: 10.1186/s12874-018-0493-y (PMC5941595; doi:10.1186/s12874-018-0493-y)
Supplement: Supplementary file 3 — Interrater agreement (Cohen’s kappa) for R-AMSTAR. (DOCX 28 kb) [file 12874_2018_493_MOESM3_ESM.docx]

**Additional file 3.** Interrater agreement (Cohen’s kappa) for R-AMSTAR.

| **Item** | **Criterion** | **Kappa** | **SEM** | **95% CI** |
| --- | --- | --- | --- | --- |
| 1. Was an 'a priori' design provided | A | 0.05  0  0.36 | 0.06  0  0.08 | -0.02-0.21  0-0  0.20-0.52 |
|  | B |  |  |  |
|  | C |  |  |  |
| 2. Was there duplicate study selection and data extraction | A | 0.56  0.47  0.46 | 0.10  0.09  0.09 | 0.36-0.77  0.29-0.65  0.28-0.64 |
|  | B |  |  |  |
|  | C |  |  |  |
| 3. Was a comprehensive literature search performed | A | 0.38  0.26  0.10  0.18  0.50 | 0.28  0.23  0.05  0.07  0.12 | -0.17-0.94  -0.19-0.71  0.01-0.19  0.04-0.33  0.25-0.75 |
|  | B |  |  |  |
|  | C |  |  |  |
|  | D |  |  |  |
|  | E |  |  |  |
| 4. Was the status of publication (i.e. grey literature) used as an inclusion criterion | A | 0.06  -0.16  0.23  0.78 | 0.06  0.08  0.09  0.07 | -0.06-0.18  -0.32-0.00  0.04-0.41  0.62-0.88 |
|  | B |  |  |  |
|  | C |  |  |  |
|  | D |  |  |  |
| 5. Was a list of studies (included and excluded) provided | A | 0.80  0.79  0.34  0.48 | 0.10  0.06  0.08  0.09 | 0.60-0.99  0.67-0.92  0.18-0.50  0.30-0.66 |
|  | B |  |  |  |
|  | C |  |  |  |
|  | D |  |  |  |
| 6. Were the characteristics of the included studies provided | A | 0.62  0.45  0.53 | 0.09  0.08  0.08 | 0.44-0.80  0.29-0.61  0.36-0.70 |
|  | B |  |  |  |
|  | C |  |  |  |
| 7. Was the scientific quality of the included studies assessed and documented | A | 0.37  0.47  0.37  0.41 | 0.15  0.10  0.11  0.11 | 0.08-0.66  0.27-0.67  0.17-0.58  0.18-0.64 |
|  | B |  |  |  |
|  | C |  |  |  |
|  | D |  |  |  |
| 8. Was the scientific quality of the included studies used appropriately in formulating conclusions | A | 0.54  0.26  0.30  0.40 | 0.09  0.09  0.07  0.27 | 0.36-0.72  0.08-0.43  0.16-0.45  -0.16-0.94 |
|  | B |  |  |  |
|  | C |  |  |  |
|  | D |  |  |  |
| 9. Were the methods used to combine the findings of studies appropriate | A | 0.55  0.72  0.56  0.67  0.08 | 0.09  0.07  0.09  0.08  0.06 | 0.37-0.74  0.57-0.86  0.39-0.74  0.52-0.82  -0.04-0.20 |
|  | B |  |  |  |
|  | C |  |  |  |
|  | D |  |  |  |
|  | E |  |  |  |
| 10. Was the likelihood of publication bias assessed | A | 0.56  0.66  0.78 | 0.08  0.09  0.11 | 0.41-0.72  0.48-0.84  0.56-0.99 |
|  | B |  |  |  |
|  | C |  |  |  |
| 11. Was the conflict of interest included | A | 0.58  0.57  0.46 | 0.09  0.08  0.13 | 0.40-0.75  0.41-0.73  0.20-0.73 |
|  | B |  |  |  |
|  | C |  |  |  |
| Overall agreement (mean score of 41 items) | | 0.43 | 0.04 | 0.36-0.50 |

**Abbreviations:** R-AMSTAR, revised version of Assessment of Multiple Systematic Reviews checklist; SEM, standard error of the mean; CI, confidence interval.
