# Supplementary material for: Comparison of methodological quality rating of systematic reviews on neuropathic pain using AMSTAR and R-AMSTAR
Source: BMC Med Res Methodol. 2018 May 8;18:37. doi: 10.1186/s12874-018-0493-y (PMC5941595; doi:10.1186/s12874-018-0493-y)
Supplement: Supplementary file 4 — References of included studies. (DOCX 139 kb) [file 12874_2018_493_MOESM4_ESM.docx]

**Additional file 4.** List of included studies.

| **Study** |
| --- |
| Wolff RF, 2010 [[1](#_ENREF_1)] |
| Wolff RF, 2011 [[2](#_ENREF_2)] |
| Chalk C, 2007 [[3](#_ENREF_3)] |
| Mijnhout GS, 2012 [[4](#_ENREF_4)] |
| Hempenstall K, 2005 [[5](#_ENREF_5)] |
| Collins SL, 2000 [[6](#_ENREF_6)] |
| Gutierrez-Alvarez AM, 2007 [[7](#_ENREF_7)] |
| Chen W, 2013 a [[8](#_ENREF_8)] |
| Hao C, 2013 [[9](#_ENREF_9)] |
| Xu HB, 2012 [[10](#_ENREF_10)] |
| Ney JP, 2013 [[11](#_ENREF_11)] |
| Hahne AJ, 2010 [[12](#_ENREF_12)] |
| Chaudhry V, 2008 [[13](#_ENREF_13)] |
| Vrancken AFJE, 2004 [[14](#_ENREF_14)] |
| Zambelli Pinto R, 2012 b [[15](#_ENREF_15)] |
| Jin DM, 2010 [[16](#_ENREF_16)] |
| Chen L, 2014 [[17](#_ENREF_17)] |
| Luijsterburg PAJ, 2007 [[18](#_ENREF_18)] |
| Sun Y, 2005 [[19](#_ENREF_19)] |
| Wong MC; 2007 [[20](#_ENREF_20)] |
| Fan H, 2014 [[21](#_ENREF_21)] |
| Meng FY, 2013 [[22](#_ENREF_22)] |
| Zhang SS, 2015 [[23](#_ENREF_23)] |
| Roncoroni C, 2011 [[24](#_ENREF_24)] |
| Roberts ST, 2009 [[25](#_ENREF_25)] |
| Tzellos TG, 2008 [[26](#_ENREF_26)] |
| Stein C, 2013 [[27](#_ENREF_27)] |
| Vasiliadis HS, 2014 [[28](#_ENREF_28)] |
| Zambelli Pinto R, 2012 [[29](#_ENREF_29)] |
| Finnerup NB, 2010 [[30](#_ENREF_30)] |
| Chou R, 2009 [[31](#_ENREF_31)] |
| Vrancken AFJE, 2007 [[32](#_ENREF_32)] |
| van Eijk J, 2013 [[33](#_ENREF_33)] |
| Quilici S, 2009 [[34](#_ENREF_34)] |
| Xu Q, 2013 [[35](#_ENREF_35)] |
| Deng H, 2014 [[36](#_ENREF_36)] |
| Thoma A, 2004 [[37](#_ENREF_37)] |
| Rasouli MR, 2014 [[38](#_ENREF_38)] |
| Seidel S, 2009 [[39](#_ENREF_39)] |
| Zhang J, 2013 [[40](#_ENREF_40)] |
| O'Connor D, 2003 [[41](#_ENREF_41)] |
| Sayegh ET, 2015 [[42](#_ENREF_42)] |
| Sommer C, 2015 [[43](#_ENREF_43)] |
| Zhou M, 2013 [[44](#_ENREF_44)] |
| Alviar MJM, 2011 [[45](#_ENREF_45)] |
| Phillips TJ, 2010 [[46](#_ENREF_46)] |
| Alves TCA, 2004 [[47](#_ENREF_47)] |
| Hurley RW, 2008 [[48](#_ENREF_48)] |
| Bansal D, 2013 [[49](#_ENREF_49)] |
| Jacobs WCH, 2011 [[50](#_ENREF_50)] |
| Jacobs WCH, 2012 [[51](#_ENREF_51)] |
| Scholten RJPM, 2007 [[52](#_ENREF_52)] |
| Verdugo RJ, 2008 [[53](#_ENREF_53)] |
| Snedecor SJ, 2013b [[54](#_ENREF_54)] |
| Edelsberg JS, 2011 [[55](#_ENREF_55)] |
| Snedecor SJ, 2014 [[56](#_ENREF_56)] |
| Snedecor SJ, 2014 [[57](#_ENREF_57)] |
| Page MJ, 2012 b [[58](#_ENREF_58)] |
| Derry S, 2013 [[59](#_ENREF_59)] |
| Derry S, 2012b [[60](#_ENREF_60)] |
| Chen W, 2011 [[61](#_ENREF_61)] |
| Wang Q, 2011 [[62](#_ENREF_62)] |
| Mulvey MR, 2010 [[63](#_ENREF_63)] |
| Quraishi NA, 2012 [[64](#_ENREF_64)] |
| Alper BS, 2002 [[65](#_ENREF_65)] |
| Volmink J, 1996 [[66](#_ENREF_66)] |
| Zhang W, 2013 [[67](#_ENREF_67)] |
| Thoomes EJ, 2013 [[68](#_ENREF_68)] |
| Edelsberg J, 2011 [[69](#_ENREF_69)] |
| Boychuk DG, 2015 [[70](#_ENREF_70)] |
| Liu H, 2010 [[71](#_ENREF_71)] |
| Rudroju N, 2013 [[72](#_ENREF_72)] |
| O’Connor D, 2012 [[73](#_ENREF_73)] |
| Chan YC, 2012 [[74](#_ENREF_74)] |
| Thomson CE, 2004 [[75](#_ENREF_75)] |
| Watson CPN, 2010 [[76](#_ENREF_76)] |
| Leung A, 2009 [[77](#_ENREF_77)] |
| Plaghki L, 2004 [[78](#_ENREF_78)] |
| Smith N, 2013 [[79](#_ENREF_79)] |
| van Alfen N, 2009 [[80](#_ENREF_80)] |
| DePalma MJ, 2005 [[81](#_ENREF_81)] |
| Zlowodzki M, 2007 [[82](#_ENREF_82)] |
| Ricard E, 2013 [[83](#_ENREF_83)] |
| Dasenbrock HH, 2012 [[84](#_ENREF_84)] |
| Watts RW, 1995 [[85](#_ENREF_85)] |
| Zakrzewska JM, 2011 [[86](#_ENREF_86)] |
| Moreno CB, 2010 [[87](#_ENREF_87)] |
| Caliandro P, 2012 [[88](#_ENREF_88)] |
| Vroomen PCAJ, 2000 [[89](#_ENREF_89)] |
| Page MJ, 2012 [[90](#_ENREF_90)] |
| Zhu Y, 2011 [[91](#_ENREF_91)] |
| Hearn L, 2014 [[92](#_ENREF_92)] |
| Wiffen PJ, 2014 b [[93](#_ENREF_93)] |
| Derry S, 2015 [[94](#_ENREF_94)] |
| Derry S, 2014 [[95](#_ENREF_95)] |
| Moore RA, 2015 [[96](#_ENREF_96)] |
| Hearn L, 2014 b [[97](#_ENREF_97)] |

**References**

1. Wolff RF, Bala MM, Westwood M, Kessels AG, Kleijnen J: **5% lidocaine medicated plaster in painful diabetic peripheral neuropathy (DPN): a systematic review**. *Swiss Med Wkly* 2010, **140**(21-22):297-306.

2. Wolff RF, Bala MM, Westwood M, Kessels AG, Kleijnen J: **5% lidocaine-medicated plaster vs other relevant interventions and placebo for post-herpetic neuralgia (PHN): a systematic review**. *Acta Neurol Scand* 2011, **123**(5):295-309.

3. Chalk C, Benstead TJ, Moore F: **Aldose reductase inhibitors for the treatment of diabetic polyneuropathy**. *Cochrane Database Syst Rev* 2007, **4**:CD004572.

4. Mijnhout GS, Kollen BJ, Alkhalaf A, Kleefstra N, Bilo HJG: **Alpha lipoic Acid for symptomatic peripheral neuropathy in patients with diabetes: a meta-analysis of randomized controlled trials**. *Int J Endocrinol* 2012, **2012**:456279.

5. Hempenstall K, Nurmikko TJ, Johnson RW, A'Hern RP, Rice AS: **Analgesic therapy in postherpetic neuralgia: a quantitative systematic review**. *PLoS medicine* 2005, **2**(7):e164.

6. Collins SL, Moore RA, McQuay HJ, Wiffen P: **Antidepressants and anticonvulsants for diabetic neuropathy and postherpetic neuralgia: a quantitative systematic review**. *J Pain Symptom Manage* 2000, **20**(6):449-458.

7. Gutierrez-Alvarez AM, Beltran-Rodriguez J, Moreno CB: **Antiepileptic drugs in treatment of pain caused by diabetic neuropathy**. *J Pain Symptom Manage* 2007, **34**(2):201-208.

8. Chen W, Zhang Y, Li X, Yang G, Liu JP: **Chinese herbal medicine for diabetic peripheral neuropathy**. *Cochrane Database Syst Rev* 2013, **10**:CD007796.

9. Hao C-z, Wu F, Lu L, Wang J, Guo Y, Liu A-j, Liao W-j, Zheng G-q: **Chinese herbal medicine for diabetic peripheral neuropathy: an updated meta-analysis of 10 high-quality randomized controlled studies**. *PLoS One* 2013, **8**(10):e76113.

10. Xu H-B, Jiang R-H, Chen X-Z, Li L: **Chinese herbal medicine in treatment of diabetic peripheral neuropathy: a systematic review and meta-analysis**. *J Ethnopharmacol* 2012, **143**(2):701-708.

11. Ney JP, Devine EB, Watanabe JH, Sullivan SD: **Comparative Efficacy of Oral Pharmaceuticals for the Treatment of Chronic Peripheral Neuropathic Pain: Meta-Analysis and Indirect Treatment Comparisons**. *Pain Med* 2013, **14**(5):706-719.

12. Hahne AJ, Ford JJ, McMeeken JM: **Conservative management of lumbar disc herniation with associated radiculopathy: a systematic review**. *Spine* 2010, **35**(11):E488-504.

13. Chaudhry V, Russell J, Belzberg A: **Decompressive surgery of lower limbs for symmetrical diabetic peripheral neuropathy**. *Cochrane Database Syst Rev* 2008, **3**:CD006152.

14. Vrancken AFJE, van Schaik IN, Hughes RAC, Notermans NC: **Drug therapy for chronic idiopathic axonal polyneuropathy**. *Cochrane Database Syst Rev* 2004, **2**:CD003456.

15. Zambelli Pinto R, Maher CG, Ferreira ML, Ferreira PH, Hancock M, Oliveira VC, McLachlan AJ, Koes B: **Drugs for relief of pain in patients with sciatica: systematic review and meta-analysis**. *BMJ* 2012, **344**:e497.

16. Jin DM, Xu Y, Geng DF, Yan TB: **Effect of transcutaneous electrical nerve stimulation on symptomatic diabetic peripheral neuropathy: a meta-analysis of randomized controlled trials**. *Diabetes Res Clin Pract* 2010, **89**(1):10-15.

17. Chen L, Duan X, Huang X, Lv J, Peng K, Xiang Z: **Effectiveness and safety of endoscopic versus open carpal tunnel decompression**. *Arch Orthop Trauma Surg* 2014, **134**(4):585-593.

18. Luijsterburg PAJ, Verhagen AP, Ostelo RWJG, van Os TAG, Peul WC, Koes BW: **Effectiveness of conservative treatments for the lumbosacral radicular syndrome: a systematic review**. *Eur Spine J* 2007, **16**(7):881-899.

19. Sun Y, Lai M-S, Lu C-J: **Effectiveness of vitamin B12 on diabetic neuropathy: systematic review of clinical controlled trials**. *Acta Neurol Taiwan* 2005, **14**(2):48-54.

20. Wong M-c, Chung JWY, Wong TKS: **Effects of treatments for symptoms of painful diabetic neuropathy: systematic review**. *BMJ* 2007, **335**(7610):87.

21. Fan H, Yu W, Zhang Q, Cao H, Li J, Wang J, Shao Y, Hu X: **Efficacy and safety of gabapentin 1800 mg treatment for post-herpetic neuralgia: a meta-analysis of randomized controlled trials**. *J Clin Pharm Ther* 2014, **39**(4):334-342.

22. Meng FY, Zhang LC, Liu Y, Pan LH, Zhu M, Li CL, Li YW, Qian W, Liang R: **Efficacy and safety of gabapentin for treatment of postherpetic neuralgia: a meta-analysis of randomized controlled trials**. *Minerva Anestesiol* 2014, **80**(5):556-567.

23. Zhang SS, Wu Z, Zhang LC, Zhang Z, Chen RP, Huang YH, Chen H: **Efficacy and safety of pregabalin for treating painful diabetic peripheral neuropathy: a meta-analysis**. *Acta Anaesthesiol Scand* 2015, **59**(2):147-159.

24. Roncoroni C, Baillet A, Durand M, Gaudin P, Juvin R: **Efficacy and tolerance of systemic steroids in sciatica: a systematic review and meta-analysis**. *Rheumatology (Oxford)* 2011, **50**(9):1603-1611.

25. Roberts ST, Willick SE, Rho ME, Rittenberg JD: **Efficacy of lumbosacral transforaminal epidural steroid injections: a systematic review**. *PM R* 2009, **1**(7):657-668.

26. Tzellos TG, Papazisis G, Amaniti E, Kouvelas D: **Efficacy of pregabalin and gabapentin for neuropathic pain in spinal-cord injury: an evidence-based evaluation of the literature**. *Eur J Clin Pharmacol* 2008, **64**(9):851-858.

27. Stein C, Eibel B, Sbruzzi G, Lago PD, Plentz RDM: **Electrical stimulation and electromagnetic field use in patients with diabetic neuropathy: systematic review and meta-analysis**. *Braz J Phys Ther* 2013, **17**(2):93-104.

28. Vasiliadis HS, Georgoulas P, Shrier I, Salanti G, Scholten RJPM: **Endoscopic release for carpal tunnel syndrome**. *Cochrane Database Syst Rev* 2014, **1**:CD008265.

29. Zambelli Pinto R, Maher CG, Ferreira ML, Hancock M, Oliveira VC, McLachlan AJ, Koes B, Ferreira PH: **Epidural corticosteroid injections in the management of sciatica: a systematic review and meta-analysis**. *Ann Intern Med* 2012, **157**(12):865-877.

30. Finnerup NB, Sindrup SH, Jensen TS: **The evidence for pharmacological treatment of neuropathic pain.** *Pain* 2010, **150**(3):573-581.

31. Chou R, Carson S, Chan BKS: **Gabapentin versus tricyclic antidepressants for diabetic neuropathy and post-herpetic neuralgia: discrepancies between direct and indirect meta-analyses of randomized controlled trials**. *J Gen Intern Med* 2009, **24**(2):178-188.

32. Vrancken AFJE, Hughes RAC, Said G, Wokke JHJ, Notermans NC: **Immunosuppressive treatment for non-systemic vasculitic neuropathy**. *Cochrane Database Syst Rev* 2007, **1**:CD006050.

33. van Eijk J, Chan YC, Russell JW: **Immunotherapy for idiopathic lumbosacral plexopathy**. *Cochrane Database Syst Rev* 2013, **12**:CD009722.

34. Quilici S, Chancellor J, Löthgren M, Simon D, Said G, Le TK, Garcia-Cebrian A, Monz B: **Meta-analysis of duloxetine vs. pregabalin and gabapentin in the treatment of diabetic peripheral neuropathic pain**. *BMC Neurol* 2009, **9**(6):1-14.

35. Xu Q, Pan J, Yu J, Liu X, Liu L, Zuo X, Wu P, Deng H, Zhang J, Ji A: **Meta-analysis of methylcobalamin alone and in combination with lipoic acid in patients with diabetic peripheral neuropathy**. *Diabetes Res Clin Pract* 2013, **101**(2):99-105.

36. Deng H, Yin J, Zhang J, Xu Q, Liu X, Liu L, Wu Z, Ji A: **Meta-analysis of methylcobalamin alone and in combination with prostaglandin E1 in the treatment of diabetic peripheral neuropathy**. *Endocrine* 2014, **46**(3):445-454.

37. Thoma A, Veltri K, Haines T, Duku E: **A meta-analysis of randomized controlled trials comparing endoscopic and open carpal tunnel decompression**. *Plast Reconstr Surg* 2004, **114**(5):1137-1146.

38. Rasouli MR, Rahimi-Movaghar V, Shokraneh F, Moradi-Lakeh M, Chou R: **Minimally invasive discectomy versus microdiscectomy/open discectomy for symptomatic lumbar disc herniation**. *Cochrane Database Syst Rev* 2014, **9**:CD010328.

39. Seidel S, Kasprian G, Sycha T, Auff E: **[Mirror therapy for phantom limb pain--a systematic review]**. *Wien Klin Wochenschr* 2009, **121**(13-14):440-444.

40. Zhang J, Yang M, Zhou M, He L, Chen N, Zakrzewska JM: **Non-antiepileptic drugs for trigeminal neuralgia**. *Cochrane Database Syst Rev* 2013, **12**:CD004029.

41. O'Connor D, Marshall SC, Massy-Westropp N, Pitt V: **Non-surgical treatment (other than steroid injection) for carpal tunnel syndrome**. *Cochrane Database Syst Rev* 2003, **1**:CD003219.

42. Sayegh ET, Strauch RJ: **Open versus Endoscopic Carpal Tunnel Release: A Meta-analysis of Randomized Controlled Trials**. *Clin Orthop Relat Res* 2015, **473**(3):1120-1132.

43. Sommer C, Welsch P, Klose P, Schaefert R, Petzke F, Hauser W: **[Opioids in chronic neuropathic pain : A systematic review and meta-analysis of efficacy, tolerability and safety in randomized placebo-controlled studies of at least 4 weeks duration]**. *Schmerz* 2015, **29**(1):35-46.

44. Zhou M, Chen N, He L, Yang M, Zhu C, Wu F: **Oxcarbazepine for neuropathic pain**. *Cochrane Database Syst Rev* 2013, **3**:CD007963.

45. Alviar MJM, Hale T, Dungca M: **Pharmacologic interventions for treating phantom limb pain**. *Cochrane Database Syst Rev* 2011, **12**:CD006380.

46. Phillips TJC, Cherry CL, Cox S, Marshall SJ, Rice ASC: **Pharmacological treatment of painful HIV-associated sensory neuropathy: a systematic review and meta-analysis of randomised controlled trials**. *PLoS One* 2010, **5**(12):e14433.

47. Alves TCA, Azevedo GS, Carvalho ESd: **[Pharmacological treatment of trigeminal neuralgia: systematic review and metanalysis.]**. *Rev Bras Anestesiol* 2004, **54**(6):836-849.

48. Hurley RW, Lesley MR, Adams MCB, Brummett CM, Wu CL: **Pregabalin as a treatment for painful diabetic peripheral neuropathy: a meta-analysis**. *Reg Anesth Pain Med* 2008, **33**(5):389-394.

49. Bansal D, Badhan Y, Gudala K, Schifano F: **Ruboxistaurin for the treatment of diabetic peripheral neuropathy: a systematic review of randomized clinical trials**. *Diabetes Metab J* 2013, **37**(5):375-384.

50. Jacobs WCH, van Tulder M, Arts M, Rubinstein SM, van Middelkoop M, Ostelo R, Verhagen A, Koes B, Peul WC: **Surgery versus conservative management of sciatica due to a lumbar herniated disc: a systematic review**. *Eur Spine J* 2011, **20**(4):513-522.

51. Jacobs WCH, Arts MP, van Tulder MW, Rubinstein SM, van Middelkoop M, Ostelo RW, Verhagen AP, Koes BW, Peul WC: **Surgical techniques for sciatica due to herniated disc, a systematic review**. *Eur Spine J* 2012, **21**(11):2232-2251.

52. Scholten RJPM, Mink van der Molen A, Uitdehaag BMJ, Bouter LM, de Vet HCW: **Surgical treatment options for carpal tunnel syndrome**. *Cochrane Database Syst Rev* 2007, **4**:CD003905.

53. Verdugo RJ, Salinas RA, Castillo JL, Cea JG: **Surgical versus non-surgical treatment for carpal tunnel syndrome**. *Cochrane Database Syst Rev* 2008, **4**:CD001552.

54. Snedecor SJ, Sudharshan L, Cappelleri JC, Sadosky A, Desai P, Jalundhwala YJ, Botteman M: **Systematic review and comparison of pharmacologic therapies for neuropathic pain associated with spinal cord injury**. *Journal of pain research* 2013, **6**:539-547.

55. Edelsberg JS, Lord C, Oster G: **Systematic review and meta-analysis of efficacy, safety, and tolerability data from randomized controlled trials of drugs used to treat postherpetic neuralgia**. *Ann Pharmacother* 2011, **45**(12):1483-1490.

56. Snedecor SJ, Sudharshan L, Cappelleri JC, Sadosky A, Desai P, Jalundhwala Y, Botteman M: **Systematic review and meta-analysis of pharmacological therapies for pain associated with postherpetic neuralgia and less common neuropathic conditions**. *Int J Clin Pract* 2014, **68**(7):900-918.

57. Snedecor SJ, Sudharshan L, Cappelleri JC, Sadosky A, Mehta S, Botteman M: **Systematic review and meta-analysis of pharmacological therapies for painful diabetic peripheral neuropathy**. *Pain practice : the official journal of World Institute of Pain* 2014, **14**(2):167-184.

58. Page MJ, O'Connor D, Pitt V, Massy-Westropp N: **Therapeutic ultrasound for carpal tunnel syndrome**. *Cochrane Database Syst Rev* 2013, **3**:CD009601.

59. Derry S, Rice ASC, Cole P, Tan T, Moore RA: **Topical capsaicin (high concentration) for chronic neuropathic pain in adults**. *Cochrane Database Syst Rev* 2013(2):CD007393.

60. Derry S, Moore RA: **Topical capsaicin (low concentration) for chronic neuropathic pain in adults**. *Cochrane Database Syst Rev* 2012, **9**:CD010111.

61. Chen W, Luo Y-F, Liu J-P: **Topical herbal medicine for treatment of diabetic peripheral neuropathy: a systematic review of randomized controlled trials**. *Forsch Komplementarmed* 2011, **18**(3):134-145.

62. Wang Q-p, Bai M: **Topiramate versus carbamazepine for the treatment of classical trigeminal neuralgia: A meta-analysis**. *CNS Drugs* 2011, **25**(10):847-857.

63. Mulvey MR, Bagnall A-M, Johnson MI, Marchant PR: **Transcutaneous electrical nerve stimulation (TENS) for phantom pain and stump pain following amputation in adults**. *Cochrane Database Syst Rev* 2010, **5**:CD007264.

64. Quraishi NA: **Transforaminal injection of corticosteroids for lumbar radiculopathy: systematic review and meta-analysis**. *Eur Spine J* 2012, **21**(2):214-219.

65. Alper BS, Lewis PR: **Treatment of postherpetic neuralgia: A systematic review of the literature**. *J Fam Pract* 2002, **51**(2):121-128.

66. Volmink J, Lancaster T, Gray S, Silagy C: **Treatments for postherpetic neuralgia—a systematic review of randomized controlled trials**. *Fam Pract* 1996, **13**(1):84-91.

67. Zhang WW, Li MQ, Liu L: **[Meta-analysis of gabapentin in the treatment of postherpetic neuralgia]**. *Chin J Contemp Neurol Neurosurg* 2013, **13**(9):760-765.

68. Thoomes EJ, Scholten-Peeters W, Koes B, Falla D, Verhagen AP: **The effectiveness of conservative treatment for patients with cervical radiculopathy: a systematic review**. *Clin J Pain* 2013, **29**(12):1073-1086.

69. Edelsberg J, Lord C, Oster G: **Systematic review of data from randomized controlled trials on the efficacy, safety and tolerability of drugs used to treat painful diabetic neuropathy**. *J Pain Manage* 2011(4):339-351.

70. Boychuk DG, Goddard G, Mauro G, Orellana MF: **The effectiveness of cannabinoids in the management of chronic nonmalignant neuropathic pain: a systematic review**. *J Oral Facial Pain Headache* 2015, **29**(1):7-14.

71. Liu H, Li H, Xu M, Chung KF, Zhang SP: **A systematic review on acupuncture for trigeminal neuralgia**. *Altern Ther Health Med* 2010, **16**(6):30-35.

72. Rudroju N, Bansal D, Talakokkula ST, Gudala K, Hota D, Bhansali A, Ghai B: **Comparative efficacy and safety of six antidepressants and anticonvulsants in painful diabetic neuropathy: A network meta-analysis.** *Pain Physician* 2013, **16**(6):E705-714.

73. O'Connor D, Page MJ, Marshall SC, Massy-Westropp N: **Ergonomic positioning or equipment for treating carpal tunnel syndrome**. *Cochrane Database Syst Rev* 2012, **1**:CD009600.

74. Chan YC, Lo YL, Chan ESY: **Immunotherapy for diabetic amyotrophy**. *Cochrane Database Syst Rev* 2012, **6**:CD006521.

75. Thomson CE, Gibson JNA, Martin D: **Interventions for the treatment of Morton's neuroma**. *Cochrane Database Syst Rev* 2004, **3**:CD003118.

76. Watson CPN, Gilron I, Sawynok J: **A qualitative systematic review of head-to-head randomized controlled trials of oral analgesics in neuropathic pain**. *Pain Res Manage* 2010, **15**(3):147-157.

77. Leung A, Donohue M, Xu R, Lee R, Lefaucheur J-P, Khedr EM, Saitoh Y, Andre-Obadia N, Rollnik J, Wallace M *et al*: **rTMS for suppressing neuropathic pain: a meta-analysis**. *J Pain* 2009, **10**(12):1205-1216.

78. Plaghki L, Adriaensen H, Morlion B, Lossignol D, Devulder J: **Systematic overview of the pharmacological management of postherpetic neuralgia. An evaluation of the clinical value of critically selected drug treatments based on efficacy and safety outcomes from randomized controlled studies**. *Dermatology* 2004, **208**(3):206-216.

79. Smith N, Masters J, Jensen C, Khan A, Sprowson A: **Systematic review of microendoscopic discectomy for lumbar disc herniation**. *Eur Spine J* 2013, **22**(11):2458-2465.

80. van Alfen N, van Engelen BGM, Hughes RAC: **Treatment for idiopathic and hereditary neuralgic amyotrophy (brachial neuritis)**. *Cochrane Database Syst Rev* 2009, **3**:CD006976.

81. DePalma MJ, Bhargava A, Slipman CW: **A critical appraisal of the evidence for selective nerve root injection in the treatment of lumbosacral radiculopathy**. *Arch Phys Med Rehabil* 2005, **86**(7):1477-1483.

82. Zlowodzki M, Chan S, Bhandari M, Kalliainen L, Schubert W: **Anterior transposition compared with simple decompression for treatment of cubital tunnel syndrome. A meta-analysis of randomized, controlled trials**. *J Bone Joint Surg Am* 2007, **89**(12):2591-2598.

83. Ricard E, Saule E, Barnetche T, Treves R, Vergne-Salle P, Bertin P: **Efficacité de la capsaïcine dans le traitement des douleurs neuropathiques: méta-analyse des essais randomisés contrôlés**. *Douleurs : Evaluation - Diagnostic - Traitement* 2013, **14**(6):286-291.

84. Dasenbrock HH, Juraschek SP, Schultz LR, Witham TF, Sciubba DM, Wolinsky J-P, Gokaslan ZL, Bydon A: **The efficacy of minimally invasive discectomy compared with open discectomy: a meta-analysis of prospective randomized controlled trials**. *J Neurosurg Spine* 2012, **16**(5):452-462.

85. Watts RW, Silagy CA: **A meta-analysis on the efficacy of epidural corticosteroids in the treatment of sciatica**. *Anaesth Intensive Care* 1995, **23**(5):564-569.

86. Zakrzewska JM, Akram H: **Neurosurgical interventions for the treatment of classical trigeminal neuralgia**. *Cochrane Database Syst Rev* 2011, **9**:CD007312.

87. Moreno CB, Gutiérrez-Álvarez AM: **Opioides en el manejo del dolor en la neuropatía diabética [Spanish]**. *Arch Neurocien* 2010, **15**(1):31-34.

88. Caliandro P, La Torre G, Padua R, Giannini F, Padua L: **Treatment for ulnar neuropathy at the elbow**. *Cochrane Database Syst Rev* 2012, **7**:CD006839.

89. Vroomen PCAJ, de Krom MC, Slofstra PD, Knottnerus JA: **Conservative treatment of sciatica: a systematic review**. *J Spinal Disord* 2000, **13**(6):463-469.

90. Page MJ, O'Connor D, Pitt V, Massy-Westropp N: **Exercise and mobilisation interventions for carpal tunnel syndrome**. *Cochrane Database Syst Rev* 2012, **6**:CD009899.

91. Zhu Y, Zhu LT, Li N, Li Y, Jin HZ: **Jiaji points combined with surrounding needling for the treatment of postherpetic neuralgia: a meta-analysis**. *Journal of Clinical Rehabilitative Tissue Engineering Research* 2011, **15**(11):2064-2068.

92. Hearn L, Derry S, Phillips T, Moore RA, Wiffen PJ: **Imipramine for neuropathic pain in adults**. *Cochrane Database Syst Rev* 2014, **5**:CD010769.

93. Wiffen PJ, Derry S, Moore RA, Lunn MPT: **Levetiracetam for neuropathic pain in adults**. *Cochrane Database Syst Rev* 2014, **7**:CD010943.

94. Derry S, Wiffen PJ, Aldington D, Moore RA: **Nortriptyline for neuropathic pain in adults**. *Cochrane Database Syst Rev* 2015, **1**:CD011209.

95. Derry S, Wiffen PJ, Moore RA, Quinlan J: **Topical lidocaine for neuropathic pain in adults**. *Cochrane Database Syst Rev* 2014, **7**:CD010958.

96. Moore RA, Wiffen PJ, Derry S, Lunn MPT: **Zonisamide for neuropathic pain in adults**. *Cochrane Database Syst Rev* 2015, **1**:CD011241.

97. Hearn L, Moore RA, Derry S, Wiffen PJ, Phillips T: **Desipramine for neuropathic pain in adults**. *Cochrane Database Syst Rev* 2014, **9**:CD011003.
