# Supplementary material for: Comparison of methodological quality rating of systematic reviews on neuropathic pain using AMSTAR and R-AMSTAR
Source: BMC Med Res Methodol. 2018 May 8;18:37. doi: 10.1186/s12874-018-0493-y (PMC5941595; doi:10.1186/s12874-018-0493-y)
Supplement: Supplementary file 5 — List of excluded studies with reasons. (DOCX 296 kb) [file 12874_2018_493_MOESM5_ESM.docx]

**Additional file 5.** List of excluded studies with reasons.

| **Study** | **Exclusion reason** |
| --- | --- |
| Mannix SM, 2013 [[1](#_ENREF_1)] | Not a SR/MA of RCTs |
| Rodine RJ, 2012 [[2](#_ENREF_2)] | Not a SR/MA of RCTs |
| Previnaire JG, 2009 [[3](#_ENREF_3)] | Not a SR/MA of RCTs |
| Attal N, 2009 [[4](#_ENREF_4)] | Not a SR/MA of RCTs |
| Gault D, 2009 [[5](#_ENREF_5)] | Not a SR/MA of RCTs |
| Lewis R, 2011 [[6](#_ENREF_6)] | Not a SR/MA of RCTs |
| Boyles R, 2011 [[7](#_ENREF_7)] | Not a SR/MA of RCTs |
| Mou J, 2013 [[8](#_ENREF_8)] | Not a SR/MA of RCTs |
| Jongen JLM, 2013 [[9](#_ENREF_9)] | Not a SR/MA of RCTs |
| Jadad A, 2001 [[10](#_ENREF_10)] | Not a SR/MA of RCTs |
| Jawahar R, 2013 [[11](#_ENREF_11)] | Not a SR/MA of RCTs |
| Clark JG, 2011 [[12](#_ENREF_12)] | Not a SR/MA of RCTs |
| Mehta S, 2013 [[13](#_ENREF_13)] | Not a SR/MA of RCTs |
| Mehta S, 2013b [[14](#_ENREF_14)] | Not a SR/MA of RCTs |
| Khadem T, 2013 [[15](#_ENREF_15)] | Not a SR/MA of RCTs |
| Khalil N, 2012 [[16](#_ENREF_16)] | Not a SR/MA of RCTs |
| Xia L, 2014 [[17](#_ENREF_17)] | Not a SR/MA of RCTs |
| Shamliyan TA, 2014 [[18](#_ENREF_18)] | Not a SR/MA of RCTs |
| Schemmel KE, 2010 [[19](#_ENREF_19)] | Not a SR/MA of RCTs |
| Jawahar R, 2014 [[20](#_ENREF_20)] | Not a SR/MA of RCTs |
| Guy S, 2014 [[21](#_ENREF_21)] | Not a SR/MA of RCTs |
| Goodyear-Smith F, 2009 [[22](#_ENREF_22)] | Not a SR/MA of RCTs |
| Schröder S, 2013 [[23](#_ENREF_23)] | Not a SR/MA of RCTs |
| Moreno CB, 2012 [[24](#_ENREF_24)] | Not a SR/MA of RCTs |
| Kumar B, 2009 [[25](#_ENREF_25)] | Not a SR/MA of RCTs |
| Heary RF, 2009 [[26](#_ENREF_26)] | Not a SR/MA of RCTs |
| Feuerstein M, 1999 [[27](#_ENREF_27)] | Not a SR/MA of RCTs |
| Wang YF, 2014 [[28](#_ENREF_28)] | Not a SR/MA of RCTs |
| Lewis RA, 2013 [[29](#_ENREF_29)] | Not a SR/MA of RCTs |
| Shamji MF, 2013 [[30](#_ENREF_30)] | Not a SR/MA of RCTs |
| Santee JA, 2002 [[31](#_ENREF_31)] | Not a SR/MA of RCTs |
| Soledad Cepeda M,2002 [[32](#_ENREF_32)] | Not a SR/MA of RCTs |
| Denkers MR, 2002 [[33](#_ENREF_33)] | Not a SR/MA of RCTs |
| Dirckx M, 2012 [[34](#_ENREF_34)] | Not a SR/MA of RCTs |
| Falaki F, 2014 [[35](#_ENREF_35)] | Not a SR/MA of RCTs |
| Roth T, 2010 [[36](#_ENREF_36)] | Not a SR/MA of RCTs |
| Engel A, 2014 [[37](#_ENREF_37)] | Not a SR/MA of RCTs |
| Hu X, 2014 [[38](#_ENREF_38)] | Not a SR/MA of RCTs |
| Bennett MI, 2011 [[39](#_ENREF_39)] | Not a SR/MA of RCTs |
| MacVicar J, 2013 [[40](#_ENREF_40)] | Not a SR/MA of RCTs |
| Hayek SM, 2009 [[41](#_ENREF_41)] | Not a SR/MA of RCTs |
| Manchikanti L, 2012 [[42](#_ENREF_42)] | Not a SR/MA of RCTs |
| Abbass K, 2012 [[43](#_ENREF_43)] | Not a SR/MA of RCTs |
| Freeman R, 2008 [[44](#_ENREF_44)] | Not a SR/MA of RCTs |
| Rushton DN, 2002 [[45](#_ENREF_45)] | Not a SR/MA of RCTs |
| Pieber K, 2010 [[46](#_ENREF_46)] | Not a SR/MA of RCTs |
| Ong KS, 2003 [[47](#_ENREF_47)] | Not a SR/MA of RCTs |
| Semel D, 2010 [[48](#_ENREF_48)] | Not a SR/MA of RCTs |
| Jacobs WCH, 2013 [[49](#_ENREF_49)] | Not a SR/MA of RCTs |
| Hemstreet B, 2001 [[50](#_ENREF_50)] | Not a SR/MA of RCTs |
| Goh L, 2003 [[51](#_ENREF_51)] | Not a SR/MA of RCTs |
| Watters WCI, 2009 [[52](#_ENREF_52)] | Not a SR/MA of RCTs |
| Faas A, 1996 [[53](#_ENREF_53)] | Not a SR/MA of RCTs |
| Backonja M, 2003 [[54](#_ENREF_54)] | Not a SR/MA of RCTs |
| Pavan-Langston D, 2008 [[55](#_ENREF_55)] | Not a SR/MA of RCTs |
| Goebel A, 2010 [[56](#_ENREF_56)] | Not a SR/MA of RCTs |
| Nardone R, 2014 [[57](#_ENREF_57)] | Not a SR/MA of RCTs |
| Wiedemann B, 1997 [[58](#_ENREF_58)] | Not a SR/MA of RCTs |
| Watson CPN, 2004 [[59](#_ENREF_59)] | Not a SR/MA of RCTs |
| Gan EY, 2013 [[60](#_ENREF_60)] | Not a SR/MA of RCTs |
| Bui J, 2013 [[61](#_ENREF_61)] | Not a SR/MA of RCTs |
| Manchikanti L, 2013a [[62](#_ENREF_62)] | Not a SR/MA of RCTs |
| Manchikanti L, 2013b [[63](#_ENREF_63)] | Not a SR/MA of RCTs |
| Sekula RF, 2011 [[64](#_ENREF_64)] | Not a SR/MA of RCTs |
| Kamper SJ, 2014 [[65](#_ENREF_65)] | Not a SR/MA of RCTs |
| Wijayasinghe N, 2014 [[66](#_ENREF_66)] | Not a SR/MA of RCTs |
| McKeon JMM, 2008 [[67](#_ENREF_67)] | Not a SR/MA of RCTs |
| Irving GA, 2012 [[68](#_ENREF_68)] | Not a SR/MA of RCTs |
| Watson CPN, 2011 [[69](#_ENREF_69)] | Not a SR/MA of RCTs |
| Schloss JM, 2013 [[70](#_ENREF_70)] | Not a SR/MA of RCTs |
| Chan BKB, 2011 [[71](#_ENREF_71)] | Not a SR/MA of RCTs |
| Benbow SJ, 1999 [[72](#_ENREF_72)] | Not a SR/MA of RCTs |
| Koltzenburg M, 1998 [[73](#_ENREF_73)] | Not a SR/MA of RCTs |
| Wunderlich RP, 1998 [[74](#_ENREF_74)] | Not a SR/MA of RCTs |
| Griebeler ML, 2014 [[75](#_ENREF_75)] | Not a SR/MA of RCTs |
| Watson CPN, 2002 [[76](#_ENREF_76)] | Not a SR/MA of RCTs |
| Leheup BF, 2006 [[77](#_ENREF_77)] | Not a SR/MA of RCTs |
| Mou J, 2014 [[78](#_ENREF_78)] | Not a SR/MA of RCTs |
| Lord SM, 2002 [[79](#_ENREF_79)] | Not a SR/MA of RCTs |
| Tuleasca C, 2014 [[80](#_ENREF_80)] | Not a SR/MA of RCTs |
| Abdi S, 2005 [[81](#_ENREF_81)] | Not a SR/MA of RCTs |
| Carroll DG, 2004 [[82](#_ENREF_82)] | Not a SR/MA of RCTs |
| Miller LE, 2011 [[83](#_ENREF_83)] | Not a SR/MA of RCTs |
| Lihua P, 2013 [[84](#_ENREF_84)] | Not a SR/MA of RCTs |
| Turner JA, 2004 [[85](#_ENREF_85)] | Not a SR/MA of RCTs |
| Taylor RS, 2006 [[86](#_ENREF_86)] | Not a SR/MA of RCTs |
| Fabregat G, 2013 [[87](#_ENREF_87)] | Not a SR/MA of RCTs |
| Matz PG, 2009 [[88](#_ENREF_88)] | Not a SR/MA of RCTs |
| Müller R, 2012 [[89](#_ENREF_89)] | Not a SR/MA of RCTs |
| Singh D, 2003 [[90](#_ENREF_90)] | Not a SR/MA of RCTs |
| Ziegler D, 2004 [[91](#_ENREF_91)] | Not a SR/MA of RCTs |
| Padilla M, 2000 [[92](#_ENREF_92)] | Not a SR/MA of RCTs |
| Johnson MI, 2011 [[93](#_ENREF_93)] | Not a SR/MA of RCTs |
| Ziegler D, 2004 b [[94](#_ENREF_94)] | Not a SR/MA of RCTs |
| Joss JD, 1999 [[95](#_ENREF_95)] | Not a SR/MA of RCTs |
| Laird MA, 2000 [[96](#_ENREF_96)] | Not a SR/MA of RCTs |
| Vance CGT, 2014 [[97](#_ENREF_97)] | Not a SR/MA of RCTs |
| Tatli M, 2008 [[98](#_ENREF_98)] | Not a SR/MA of RCTs |
| Andrews JC, 2011 [[99](#_ENREF_99)] | Not a SR/MA of RCTs |
| Ahn NU, 2001 [[100](#_ENREF_100)] | Not a SR/MA of RCTs |
| Cetas JS, 2008 [[101](#_ENREF_101)] | Not a SR/MA of RCTs |
| Criner TM, 1999 [[102](#_ENREF_102)] | Not a SR/MA of RCTs |
| Jimenez DF, 1998 [[103](#_ENREF_103)] | Not a SR/MA of RCTs |
| Dy SM, 2010 [[104](#_ENREF_104)] | Not a SR/MA of RCTs |
| Zakrzewska JM, 2012 [[105](#_ENREF_105)] | Not a SR/MA of RCTs |
| Lopez BC, 2004 [[106](#_ENREF_106)] | Not a SR/MA of RCTs |
| Watson CPN, 2000 [[107](#_ENREF_107)] | Not a SR/MA of RCTs |
| Freeman BJC, 2008 [[108](#_ENREF_108)] | Not a SR/MA of RCTs |
| McCormick Z, 2014 [[109](#_ENREF_109)] | Not a SR/MA of RCTs |
| Gangi A, 2011 [[110](#_ENREF_110)] | Not a SR/MA of RCTs |
| Choi S, 2011 [[111](#_ENREF_111)] | Not about efficacy or safety |
| Alentado VJ, 2014 [[112](#_ENREF_112)] | Not about efficacy or safety |
| Wood MJ, 1996 [[113](#_ENREF_113)] | Not about efficacy or safety |
| Lancaster T, 1995 [[114](#_ENREF_114)] | Not about efficacy or safety |
| Cao H, 2010 [[115](#_ENREF_115)] | Not about efficacy or safety |
| Lycka BAS, 1990 [[116](#_ENREF_116)] | Not about efficacy or safety |
| Furlan AD, 2005 [[117](#_ENREF_117)] | Not about NeP |
| Ammendolia C, 2013 [[118](#_ENREF_118)] | Not about NeP |
| Machado LAC, 2009 [[119](#_ENREF_119)] | Not about NeP |
| Henschke N, 2011 [[120](#_ENREF_120)] | Not about NeP |
| Lin JH, 2012 [[121](#_ENREF_121)] | Not about NeP |
| Botelho RV, 2012 [[122](#_ENREF_122)] | Not about NeP |
| Richman JM, 2006 [[123](#_ENREF_123)] | Not about NeP |
| Chung JWY, 2013 [[124](#_ENREF_124)] | Not about NeP |
| Overdevest GM, 2015 [[125](#_ENREF_125)] | Not about NeP |
| Zhang J, 2011 [[126](#_ENREF_126)] | Not about NeP |
| Geurts JW, 2001 [[127](#_ENREF_127)] | Not about NeP |
| Oltean H, 2014 [[128](#_ENREF_128)] | Not about NeP |
| Lunn MPT, 2012 [[129](#_ENREF_129)] | Not about NeP |
| Staal BJ, 2008 [[130](#_ENREF_130)] | Not about NeP |
| Wasiak J, 2014 [[131](#_ENREF_131)] | Not about NeP |
| Rømsing J, 2000 [[132](#_ENREF_132)] | Not about NeP |
| Yousefi-Nooraie R, 2008 [[133](#_ENREF_133)] | Not about NeP |
| Furlan AD, 2008 [[134](#_ENREF_134)] | Not about NeP |
| Patel KC, 2012 [[135](#_ENREF_135)] | Not about NeP |
| Nicolucci A, 1996 [[136](#_ENREF_136)] | Not about NeP |
| Nicholson AB, 2007 [[137](#_ENREF_137)] | Not about NeP |
| Kamper SJ, 2014 b [[138](#_ENREF_138)] | Not about NeP |
| Aggarwal VR, 2011 [[139](#_ENREF_139)] | Not about NeP |
| Straube S, 2010 [[140](#_ENREF_140)] | Not about NeP |
| Moore RA, 2004 [[141](#_ENREF_141)] | Not about NeP |
| Toms L, 2008 [[142](#_ENREF_142)] | Not about NeP |
| Toms L, 2009 [[143](#_ENREF_143)] | Not about NeP |
| Bulley S, 2009 [[144](#_ENREF_144)] | Not about NeP |
| Pagnini F, 2014 [[145](#_ENREF_145)] | Not about NeP |
| Nnoaham KE, 2014 [[146](#_ENREF_146)] | Not about NeP |
| Khadilkar A, 2008 [[147](#_ENREF_147)] | Not about NeP |
| Hadley G, 2013 [[148](#_ENREF_148)] | Not about NeP |
| Stork ACJ, 2007 [[149](#_ENREF_149)] | Not about NeP |
| Giometto B, 2012 [[150](#_ENREF_150)] | Not about NeP |
| Montano N, 2013 [[151](#_ENREF_151)] | Not about NeP |
| Carstensen M, 2010 [[152](#_ENREF_152)] | Not about NeP |
| Mark DH, 2010 [[153](#_ENREF_153)] | Not about NeP |
| Macaulay J, 2007 [[154](#_ENREF_154)] | Not about NeP |
| Paley CA, 2011 [[155](#_ENREF_155)] | NeP and conditions not currently defined as NeP |
| Saarto T, 2010 [[156](#_ENREF_156)] | NeP and conditions not currently defined as NeP |
| Straube S, 2013 [[157](#_ENREF_157)]. | NeP and conditions not currently defined as NeP |
| Chaparro LE, 2012 [[158](#_ENREF_158)] | NeP and conditions not currently defined as NeP |
| Eisenberg E, 2005 [[159](#_ENREF_159)] | NeP and conditions not currently defined as NeP |
| Koes BW, 1995 [[160](#_ENREF_160)] | NeP and conditions not currently defined as NeP |
| Eisenberg E, 2006 [[161](#_ENREF_161)] | NeP and conditions not currently defined as NeP |
| Bell RF, 2012 [[162](#_ENREF_162)] | NeP and conditions not currently defined as NeP |
| Stanton TR, 2013 [[163](#_ENREF_163)] | NeP and conditions not currently defined as NeP |
| Xing D, 2013 [[164](#_ENREF_164)] | NeP and conditions not currently defined as NeP |
| Salt E, 2011 [[165](#_ENREF_165)] | NeP and conditions not currently defined as NeP |
| McQuay HJ, 1996 [[166](#_ENREF_166)] | NeP and conditions not currently defined as NeP |
| Collins S, 2010 [[167](#_ENREF_167)] | NeP and conditions not currently defined as NeP |
| McNicol ED, 2013 [[168](#_ENREF_168)] | NeP and conditions not currently defined as NeP |
| Leininger B, 2011 [[169](#_ENREF_169)] | NeP and conditions not currently defined as NeP |
| Nikolaidis I, 2010 [[170](#_ENREF_170)] | NeP and conditions not currently defined as NeP |
| Tremont-Lukats IW, 2005 [[171](#_ENREF_171)] | NeP and conditions not currently defined as NeP |
| Duehmke RM, 2006 [[172](#_ENREF_172)] | NeP and conditions not currently defined as NeP |
| Ang CD, 2008 [[173](#_ENREF_173)] | NeP and conditions not currently defined as NeP |
| Chu SH, 2015 [[174](#_ENREF_174)] | NeP and conditions not currently defined as NeP |
| Dahm KT, 2010 [[175](#_ENREF_175)] | NeP and conditions not currently defined as NeP |
| Finnerup NB, 2005 [[176](#_ENREF_176)] | NeP and conditions not currently defined as NeP |
| Vargas-Espinosa M-L, 2012 [[177](#_ENREF_177)] | NeP and conditions not currently defined as NeP |
| Romano CL, 2012 [[178](#_ENREF_178)] | NeP and conditions not currently defined as NeP |
| Boselie TFM, 2012 [[179](#_ENREF_179)] | NeP and conditions not currently defined as NeP |
| Waseem Z, 2011 [[180](#_ENREF_180)] | NeP and conditions not currently defined as NeP |
| Trinh K, 2010 [[181](#_ENREF_181)] | NeP and conditions not currently defined as NeP |
| Kumar A, 2014 [[182](#_ENREF_182)] | NeP and conditions not currently defined as NeP |
| Walker BF, 2010 [[183](#_ENREF_183)] | NeP and conditions not currently defined as NeP |
| Van Veen NHJ, 2007 [[184](#_ENREF_184)] | NeP and conditions not currently defined as NeP |
| Van Veen NHJ, 2012 [[185](#_ENREF_185)] | NeP and conditions not currently defined as NeP |
| Sesti F, 2011 [[186](#_ENREF_186)] | NeP and conditions not currently defined as NeP |
| Gadgil N, 2012 [[187](#_ENREF_187)] | NeP and conditions not currently defined as NeP |
| Brettschneider J, 2013 [[188](#_ENREF_188)] | NeP and conditions not currently defined as NeP |
| Sultan A, 2008 [[189](#_ENREF_189)] | NeP and conditions not currently defined as NeP |
| Lunn MPT, 2014 [[190](#_ENREF_190)] | NeP and conditions not currently defined as NeP |
| Hilde G, 1998 [[191](#_ENREF_191)] | NeP and conditions not currently defined as NeP |
| Thrane S, 2013 [[192](#_ENREF_192)] | NeP and conditions not currently defined as NeP |
| Zhang WY, 1994 [[193](#_ENREF_193)] | NeP and conditions not currently defined as NeP |
| Kroeling P, 2013 [[194](#_ENREF_194)] | NeP and conditions not currently defined as NeP |
| Choi HJ, 2013 [[195](#_ENREF_195)] | NeP and conditions not currently defined as NeP |
| Carr DB, 2004 [[196](#_ENREF_196)] | NeP and conditions not currently defined as NeP |
| Blum K, 2008 [[197](#_ENREF_197)] | NeP and conditions not currently defined as NeP |
| Devulder J, 2009 [[198](#_ENREF_198)] | NeP and conditions not currently defined as NeP |
| Engers AJ, 2008 [[199](#_ENREF_199)] | NeP and conditions not currently defined as NeP |
| Noble M, 2010 [[200](#_ENREF_200)] | NeP and conditions not currently defined as NeP |
| Gross A, 2010 [[201](#_ENREF_201)] | NeP and conditions not currently defined as NeP |
| Iskedjian M, 2007 [[202](#_ENREF_202)] | NeP and conditions not currently defined as NeP |
| Lee Y-C, 2010 [[203](#_ENREF_203)] | NeP and conditions not currently defined as NeP |
| Humble SR, 2014 [[204](#_ENREF_204)] | NeP and conditions not currently defined as NeP |
| Haroutiunian S, 2012 [[205](#_ENREF_205)] | NeP and conditions not currently defined as NeP |
| Derry S, 2012 [[206](#_ENREF_206)] | NeP and conditions not currently defined as NeP |
| O'Connell NE, 2014 [[207](#_ENREF_207)] | NeP and conditions not currently defined as NeP |
| Boldt I, 2014 [[208](#_ENREF_208)] | NeP and conditions not currently defined as NeP |
| Roelofs PDDM, 2008 [[209](#_ENREF_209)] | NeP and conditions not currently defined as NeP |
| McNicol ED, 2005 [[210](#_ENREF_210)] | NeP and conditions not currently defined as NeP |
| Chaparro LE, 2013 [[211](#_ENREF_211)] | NeP and conditions not currently defined as NeP |
| Deyo RA, 2015 [[212](#_ENREF_212)] | NeP and conditions not currently defined as NeP |
| Zeppetella G, 2013 [[213](#_ENREF_213)] | NeP and conditions not currently defined as NeP |
| Kalso E, 2004 [[214](#_ENREF_214)] | NeP and conditions not currently defined as NeP |
| Welsch P, 2015 [[215](#_ENREF_215)] | NeP and conditions not currently defined as NeP |
| Wiffen PJ, 2013 [[216](#_ENREF_216)] | NeP and conditions not currently defined as NeP |
| van Tulder MW, 2006 [[217](#_ENREF_217)] | NeP and conditions not currently defined as NeP |
| Schmidt-Hansen M, 2015 [[218](#_ENREF_218)] | NeP and conditions not currently defined as NeP |
| Gaskell H, 2014 [[219](#_ENREF_219)] | NeP and conditions not currently defined as NeP |
| Liu J, 2013 [[220](#_ENREF_220)] | NeP and conditions not currently defined as NeP |
| Moore RA, 2009 [[221](#_ENREF_221)] | NeP and conditions not currently defined as NeP |
| Baidya DK, 2011 [[222](#_ENREF_222)] | NeP and conditions not currently defined as NeP |
| Vossen H, 2009 [[223](#_ENREF_223)] | NeP and conditions not currently defined as NeP |
| Jacobs W, 2004 [[224](#_ENREF_224)] | NeP and conditions not currently defined as NeP |
| Rubinstein SM, 2012 [[225](#_ENREF_225)] | NeP and conditions not currently defined as NeP |
| Rubinstein SM, 2011 [[226](#_ENREF_226)] | NeP and conditions not currently defined as NeP |
| Benyamin R, 2009 [[227](#_ENREF_227)] | NeP and conditions not currently defined as NeP |
| Martell BA, 2007 [[228](#_ENREF_228)] | NeP and conditions not currently defined as NeP |
| Granot R, 2007 [[229](#_ENREF_229)] | NeP and conditions not currently defined as NeP |
| Ebadi S, 2014 [[230](#_ENREF_230)] | NeP and conditions not currently defined as NeP |
| Jacobs W, 2012 [[231](#_ENREF_231)] | NeP and conditions not currently defined as NeP |
| Cossins L, 2013 [[232](#_ENREF_232)] | NeP and conditions not currently defined as NeP |
| Tengrungsun T, 2012 [[233](#_ENREF_233)] | NeP and conditions not currently defined as NeP |
| Rabinovitch DL, 2009 [[234](#_ENREF_234)] | NeP and conditions not currently defined as NeP |
| Lee MS, 2008 [[235](#_ENREF_235)] | NeP and conditions not currently defined as NeP |
| Morales-Osorio MA, 2012 [[236](#_ENREF_236)] | NeP and conditions not currently defined as NeP |
| White CM, 2004 [[237](#_ENREF_237)] | Pain not measured |
| Chen W, 2013 [[238](#_ENREF_238)] | Pain not measured |
| Han T, 2012 [[239](#_ENREF_239)] | Pain not measured |
| Wu J, 2014 [[240](#_ENREF_240)] | Pain not measured |
| Coulthard P, 2014 [[241](#_ENREF_241)] | Pain not measured |
| Chin YH, 2005 [[242](#_ENREF_242)] | Pain not measured |
| Huntley AL, 2005 [[243](#_ENREF_243)] | Full-text article unavailable |
| Yang LJ, 2013 [[244](#_ENREF_244)] | Full-text article unavailable |
| Gibson JNA, 2007 [[245](#_ENREF_245)] | Updated |

**Abbreviations:** SR/MA: systematic review with or without meta-analysis; RCT: randomized controlled trial; NeP: neuropathic pain.

**References**

1. Mannix SM, O'Sullivan C, Kelly GA: **Acupuncture for managing phantom-limb syndrome: a systematic review**. In: *Medical Acupuncture.* vol. 25; 2013: 23-42.

2. Rodine RJ, Vernon H: **Cervical radiculopathy: a systematic review on treatment by spinal manipulation and measurement with the Neck Disability Index**. *Journal of the Canadian Chiropractic Association* 2012, **56**(1):18-28.

3. Previnaire J-G, Nguyen JP, Perrouin-Verbe B, Fattal C: **Chronic neuropathic pain in spinal cord injury: efficiency of deep brain and motor cortex stimulation therapies for neuropathic pain in spinal cord injury patients**. *Annals of Physical & Rehabilitation Medicine* 2009, **52**(2):188-193.

4. Attal N, Mazaltarine G, Perrouin-Verbe B, Albert T, Medicine SFSfP, Rehabilitation: **Chronic neuropathic pain management in spinal cord injury patients. What is the efficacy of pharmacological treatments with a general mode of administration? (oral, transdermal, intravenous)**. *Annals of Physical & Rehabilitation Medicine* 2009, **52**(2):124-141.

5. Gault D, Morel-Fatio M, Albert T, Fattal C: **Chronic neuropathic pain of spinal cord injury: what is the effectiveness of psychocomportemental management?** *Annals of Physical & Rehabilitation Medicine* 2009, **52**(2):167-172.

6. Lewis R, Williams N, Matar H, Din N, Fitzsimmons D, Phillips C, Jones M, Sutton A, Burton K, Nafees S *et al*: **The clinical effectiveness and cost-effectiveness of management strategies for sciatica: systematic review and economic model**. *Health Technol Assess* 2011, **15**(39):1-578.

7. Boyles R, Toy P, Mellon JJ, Hayes M, Hammer B: **Effectiveness of manual physical therapy in the treatment of cervical radiculopathy: a systematic review**. *Journal of Manual & Manipulative Therapy* 2011, **19**(3):135-142.

8. Mou J, Paillard F, Turnbull B, Trudeau J, Stoker M, Katz NP: **Efficacy of Qutenza (capsaicin) 8% patch for neuropathic pain: a meta-analysis of the Qutenza Clinical Trials Database**. *Pain* 2013, **154**(9):1632-1639.

9. Jongen JLM, Huijsman ML, Jessurun J, Ogenio K, Schipper D, Verkouteren DRC, Moorman PW, van der Rijt CCD, Vissers KC: **The evidence for pharmacologic treatment of neuropathic cancer pain: beneficial and adverse effects**. *Journal of Pain & Symptom Management* 2013, **46**(4):581-590.e581.

10. Jadad A, O'Brien MA, Wingerchuk D, Angle P, Biagi H, Denkers M, Tamayo C, Gauld M: **Management of chronic central neuropathic pain following traumatic spinal cord injury**. *Evidence Report: Technology Assessment (Summary)* 2001(45):1-5.

11. Jawahar R, Oh U, Yang S, Lapane K: **A Systematic Review of Pharmacological Pain Management in Multiple Sclerosis**. *Drugs* 2013, **73**(15):1711-1722.

12. Clark JG, Abdullah KG, Steinmetz MP, Benzel EC, Mroz TE: **Minimally Invasive versus Open Cervical Foraminotomy: A Systematic Review**. *Global Spine Journal* 2011, **1**(1):9-14.

13. Mehta S, Orenczuk K, McIntyre A, Willems G, Wolfe DL, Hsieh JTC, Short C, Loh E, Teasell RW: **Neuropathic Pain Post Spinal Cord Injury Part 1: Systematic Review of Physical and Behavioral Treatment**. *Topics in Spinal Cord Injury Rehabilitation* 2013, **19**(1):61-77.

14. Mehta S, Orenczuk K, McIntyre A, Willems G, Wolfe DL, Hsieh JTC, Short C, Loh E, Teasell RW: **Neuropathic Pain Post Spinal Cord Injury Part 2: Systematic Review at Dorsal Root Entry Zone Procedure**. *Topics in Spinal Cord Injury Rehabilitation* 2013, **19**(1):78-86.

15. Khadem T, Stevens V: **Therapeutic Options for the Treatment of Postherpetic Neuralgia: A Systematic Review**. *Journal of pain & palliative care pharmacotherapy* 2013, **27**(3):268-283.

16. Khalil N, Nicotra A, Rakowicz W: **Treatment for meralgia paraesthetica**. In: *Cochrane Database of Systematic Reviews.* John Wiley & Sons, Ltd; 2012.

17. Xia L, Zhong J, Zhu J, Wang Y-N, Dou N-N, Liu M-X, Visocchi M, Li S-T: **Effectiveness and safety of microvascular decompression surgery for treatment of trigeminal neuralgia: a systematic review**. *J Craniofac Surg* 2014, **25**(4):1413-1417.

18. Shamliyan TA, Staal JB, Goldmann D, Sands-Lincoln M: **Epidural steroid injections for radicular lumbosacral pain: a systematic review**. *Phys Med Rehabil Clin N Am* 2014, **25**(2):471-489.e471-450.

19. Schemmel KE, Padiyara RS, D'Souza JJ: **Aldose reductase inhibitors in the treatment of diabetic peripheral neuropathy: a review**. *J Diabetes Complications* 2010, **24**(5):354-360.

20. Jawahar R, Oh U, Yang S, Lapane K: **Alternative approaches: a systematic review of non-pharmacological treatments for non-spastic and non-trigeminal pain in patients with multiple sclerosis (Provisional abstract)**. In: *Database of Abstracts of Reviews of Effects.* 2014: epub.

21. Guy S, Mehta S, Leff L, Teasell R, Loh E: **Anticonvulsant medication use for the management of pain following spinal cord injury: systematic review and effectiveness analysis**. *Spinal Cord* 2014, **52**(2):89-96.

22. Goodyear-Smith F, Halliwell J: **Anticonvulsants for neuropathic pain: gaps in the evidence**. *Clin J Pain* 2009, **25**(6):528-536.

23. Schröder S, Beckmann K, Franconi G, Meyer-Hamme G, Friedemann T, Greten HJ, Rostock M, Efferth T: **Can medical herbs stimulate regeneration or neuroprotection and treat neuropathic pain in chemotherapy-induced peripheral neuropathy?** *Evidence-Based Complementary & Alternative Medicine: eCAM* 2013, **2013**:423713.

24. Moreno CB, Hernandez-Beltran N, Munevar D, Gutierrez-Alvarez AM: **Central neuropathic pain in Parkinson's disease**. *Neurologia* 2012, **27**(8):500-503.

25. Kumar B, Kalita J, Kumar G, Misra UK: **Central poststroke pain: a review of pathophysiology and treatment**. *Anesth Analg* 2009, **108**(5):1645-1657.

26. Heary RF, Ryken TC, Matz PG, Anderson PA, Groff MW, Holly LT, Kaiser MG, Mummaneni PV, Choudhri TF, Vresilovic EJ *et al*: **Cervical laminoforaminotomy for the treatment of cervical degenerative radiculopathy**. *Journal of Neurosurgery Spine* 2009, **11**(2):198-202.

27. Feuerstein M, Burrell LM, Miller VI, Lincoln A, Huang GD, Berger R: **Clinical management of carpal tunnel syndrome: a 12-year review of outcomes**. *Am J Ind Med* 1999, **35**(3):232-245.

28. Wang YF, Chen PY, Chang W, Zhu FQ, Xu LL, Wang SL, Chang LY, Luo J, Liu GJ: **Clinical significance of tumor necrosis factor-α inhibitors in the treatment of sciatica: a systematic review and meta-analysis (Provisional abstract)**. In: *Database of Abstracts of Reviews of Effects.* 2014: e103147.

29. Lewis RA, Williams NH, Sutton AJ, Burton K, Ud Din N, Matar HE, Hendry M, Phillips CJ, Nafees S, Fitzsimmons D *et al*: **Comparative clinical effectiveness of management strategies for sciatica: systematic review and network meta-analyses (Provisional abstract)**. In: *Spine Journal.* 2013: epub.

30. Shamji MF, Massicotte EM, Traynelis VC, Norvell DC, Hermsmeyer JT, Fehlings MG: **Comparison of anterior surgical options for the treatment of multilevel cervical spondylotic myelopathy: a systematic review**. *Spine* 2013, **38**(22):S195-209.

31. Santee JA: **Corticosteroids for herpes zoster: what do they accomplish? (Structured abstract)**. In: *American Journal of Clinical Dermatology.* 2002: 517-524.

32. Soledad Cepeda M, Lau J, Carr DB: **Defining the therapeutic role of local anesthetic sympathetic blockade in complex regional pain syndrome: A narrative and systematic review**. *The Clinical Journal of Pain* 2002, **18**(4):216-233.

33. Denkers MR, Biagi HL, O'Brien MA, Jadad AR, Gauld ME: **Dorsal root entry zone lesioning used to treat central neuropathic pain in patients with traumatic spinal cord injury: a systematic review**. *Spine* 2002, **27**(7):E177-184.

34. Dirckx M, Stronks DL, Groeneweg G, Huygen FJ: **Effect of immunomodulating medications in complex regional pain syndrome: a systematic review**. *Clin J Pain* 2012, **28**(4):355-363.

35. Falaki F, Nejat AH, Dalirsani Z: **The Effect of Low-level Laser Therapy on Trigeminal Neuralgia: A Review of Literature**. *Journal of dental research, dental clinics, dental prospects* 2014, **8**(1):1-5.

36. Roth T, van Seventer R, Murphy TK: **The effect of pregabalin on pain-related sleep interference in diabetic peripheral neuropathy or postherpetic neuralgia: a review of nine clinical trials**. *Curr Med Res Opin* 2010, **26**(10):2411-2419.

37. Engel A, King W, MacVicar J, Standards Division of the International Spine Intervention S: **The effectiveness and risks of fluoroscopically guided cervical transforaminal injections of steroids: a systematic review with comprehensive analysis of the published data**. *Pain Medicine* 2014, **15**(3):386-402.

38. Hu X, Trevelyan E, Yang G, Lee MS, Lorenc A, Liu J, Robinson N: **The effectiveness of acupuncture/TENS for phantom limb syndrome. I: a systematic review of controlled clinical trials (Provisional abstract)**. In: *Database of Abstracts of Reviews of Effects.* 2014: 355-364.

39. Bennett Ml: **Effectiveness of antiepileptic or antidepressant drugs when added to opioids for cancer pain: Systematic review**. *Palliat Med* 2011, **25**(5):553-559.

40. MacVicar J, King W, Landers MH, Bogduk N: **The effectiveness of lumbar transforaminal injection of steroids: a comprehensive review with systematic analysis of the published data**. *Pain Medicine* 2013, **14**(1):14-28.

41. Hayek SM, Helm S, Benyamin RM, Singh V, Bryce DA, Smith HS: **Effectiveness of spinal endoscopic adhesiolysis in post lumbar surgery syndrome: a systematic review**. *Pain Physician* 2009, **12**(2):419-435.

42. Manchikanti L, Buenaventura RM, Manchikanti KN, Ruan X, Gupta S, Smith HS, Christo PJ, Ward SP: **Effectiveness of therapeutic lumbar transforaminal epidural steroid injections in managing lumbar spinal pain**. *Pain Physician* 2012, **15**(3):E199-245.

43. Abbass K: **Efficacy of gabapentin for treatment of adults with phantom limb pain**. *Ann Pharmacother* 2012, **46**(12):1707-1711.

44. Freeman R, Durso-Decruz E, Emir B: **Efficacy, safety, and tolerability of pregabalin treatment for painful diabetic peripheral neuropathy: findings from seven randomized, controlled trials across a range of doses**. *Diabetes Care* 2008, **31**(7):1448-1454.

45. Rushton D: **Electrical stimulation in the treatment of pain**. *Disabil Rehabil* 2002, **24**(8):407-415.

46. Pieber K, Herceg M, Paternostro-Sluga T: **Electrotherapy for the treatment of painful diabetic peripheral neuropathy: a review**. *Journal of Rehabilitation Medicine* 2010, **42**(4):289-295.

47. Ong KS, Keng SB: **Evaluation of surgical procedures for trigeminal neuralgia**. *Anesth Prog* 2003, **50**(4):181-188.

48. Semel D, Murphy TK, Zlateva G, Cheung R, Emir B: **Evaluation of the safety and efficacy of pregabalin in older patients with neuropathic pain: results from a pooled analysis of 11 clinical studies**. *BMC family practice* 2010, **11**:85.

49. Jacobs WCH, Rubinstein SM, Koes B, van Tulder MW, Peul WC: **Evidence for surgery in degenerative lumbar spine disorders**. *Best Practice & Research in Clinical Rheumatology* 2013, **27**(5):673-684.

50. Hemstreet B, Lapointe M: **Evidence for the use of gabapentin in the treatment of diabetic peripheral neuropathy**. *Clin Ther* 2001, **23**(4):520-531.

51. Goh L, Bawendi A, Samanta J, Samanta A: **An evidence-based approach to the management of low back pain and sciatica: how the evidence is applied in clinical cases**. *Musculoskeletal Care* 2003, **1**(2):119-130.

52. Watters WCI, McGirt MJ: **An evidence-based review of the literature on the consequences of conservative versus aggressive discectomy for the treatment of primary disc herniation with radiculopathy**. *Spine Journal: Official Journal of the North American Spine Society* 2009, **9**(3):240-257.

53. Faas A: **Exercises: which ones are worth trying, for which patients, and when? (Structured abstract)**. In: *Spine.* 1996: 2874-2878.

54. Backonja M, Glanzman RL: **Gabapentin dosing for neuropathic pain: evidence from randomized, placebo-controlled clinical trials**. *Clin Ther* 2003, **25**(1):81-104.

55. Pavan-Langston D: **Herpes zoster antivirals and pain management**. *Ophthalmology* 2008, **115**(2 Suppl):S13-20.

56. Goebel A: **Immunoglobulin responsive chronic pain**. *J Clin Immunol* 2010, **30 Suppl 1**:S103-108.

57. Nardone R, Holler Y, Leis S, Holler P, Thon N, Thomschewski A, Golaszewski S, Brigo F, Trinka E: **Invasive and non-invasive brain stimulation for treatment of neuropathic pain in patients with spinal cord injury: a review**. *J Spinal Cord Med* 2014, **37**(1):19-31.

58. Wiedemann B: **[Ketamine for treatment of chronic pain: meta-analysis]**. *Schmerz (Berlin, Germany)* 1997, **11**(4):276-281.

59. Watson CPN: **Management issues of neuropathic trigeminal pain from a medical perspective**. *J Orofac Pain* 2004, **18**(4):366-373.

60. Gan EY, Tian EAL, Tey HL: **Management of herpes zoster and post-herpetic neuralgia**. *American Journal of Clinical Dermatology* 2013, **14**(2):77-85.

61. Bui J, Bogduk N: **A systematic review of the effectiveness of CT-guided, lumbar transforaminal injection of steroids**. *Pain Medicine* 2013, **14**(12):1860-1865.

62. Manchikanti L, Falco FJE, Benyamin RM, Caraway DL, Deer TR, Singh V, Hameed H, Hirsch JA: **An update of the systematic assessment of mechanical lumbar disc decompression with nucleoplasty**. *Pain Physician* 2013, **16**(2 Suppl):SE25-54.

63. Manchikanti L, Singh V, Falco FJE, Calodney AK, Onyewu O, Helm S, 2nd, Benyamin RM, Hirsch JA: **An updated review of automated percutaneous mechanical lumbar discectomy for the contained herniated lumbar disc**. *Pain Physician* 2013, **16**(2 Suppl):SE151-184.

64. Sekula RF, Frederickson AM, Jannetta PJ, Quigley MR, Aziz KM, Arnone GD: **Microvascular decompression for elderly patients with trigeminal neuralgia: a prospective study and systematic review with meta-analysis**. *J Neurosurg* 2011, **114**(1):172-179.

65. Kamper SJ, Ostelo RWJG, Rubinstein SM, Nellensteijn JM, Peul WC, Arts MP, van Tulder MW: **Minimally invasive surgery for lumbar disc herniation: a systematic review and meta-analysis**. *Eur Spine J* 2014, **23**(5):1021-1043.

66. Wijayasinghe N, Andersen KG, Kehlet H: **Neural blockade for persistent pain after breast cancer surgery**. *Reg Anesth Pain Med* 2014, **39**(4):272-278.

67. McKeon JMM, Yancosek KE: **Neural gliding techniques for the treatment of carpal tunnel syndrome: a systematic review**. *J Sport Rehabil* 2008, **17**(3):324-341.

68. Irving GA, Backonja M, Rauck R, Webster LR, Tobias JK, Vanhove GF: **NGX-4010, a capsaicin 8% dermal patch, administered alone or in combination with systemic neuropathic pain medications, reduces pain in patients with postherpetic neuralgia**. *Clin J Pain* 2012, **28**(2):101-107.

69. Watson CPN, Gilron I, Sawynok J, Lynch ME: **Nontricyclic antidepressant analgesics and pain: Are serotonin norepinephrine reuptake inhibitors (SNRIs) any better?** *Pain* 2011, **152**(10):2206-2210.

70. Schloss JM, Colosimo M, Airey C, Masci PP, Linnane AW, Vitetta L: **Nutraceuticals and chemotherapy induced peripheral neuropathy (CIPN): a systematic review**. *Clin Nutr* 2013, **32**(6):888-893.

71. Chan BKB, Tam LK, Wat CY, Chung YF, Tsui SL, Cheung CW: **Opioids in chronic non-cancer pain**. *Expert opinion on pharmacotherapy* 2011, **12**(5):705-720.

72. Benbow SJ, Cossins L, MacFarlane IA: **Painful diabetic neuropathy**. *Diabet Med* 1999, **16**(8):632-644.

73. Koltzenburg M: **Painful neuropathies**. *Curr Opin Neurol* 1998, **11**(5):515-521.

74. Wunderlich RP, Peters EJ, Bosma J, Armstrong DG: **Pathophysiology and treatment of painful diabetic neuropathy of the lower extremity**. *South Med J* 1998, **91**(10):894-898.

75. Griebeler ML, Morey-Vargas OL, Brito JP, Tsapas A, Wang Z, Carranza Leon BG, Phung OJ, Montori VM, Murad MH: **Pharmacologic interventions for painful diabetic neuropathy: An umbrella systematic review and comparative effectiveness network meta-analysis**. *Ann Intern Med* 2014, **161**(9):639-649.

76. Watson CPN, Oaklander AL: **Postherpetic Neuralgia**. *Pain Practice* 2002, **2**(4):295-307.

77. Leheup BF: **Pregabaline et traitement des douleurs neuropathiques: revue de la litterature [Pregabalin for the treatment of neuropathic pain: selected review of the literature] (Provisional abstract)**. In: *Douleurs.* 2006: 304-311.

78. Mou J, Paillard F, Turnbull B, Trudeau J, Stoker M, Katz NP: **Qutenza (capsaicin) 8% patch onset and duration of response and effects of multiple treatments in neuropathic pain patients**. *Clin J Pain* 2014, **30**(4):286-294.

79. Lord SM, Bogduk N: **Radiofrequency procedures in chronic pain**. *Best Practice & Research Clinical Anaesthesiology* 2002, **16**(4):597-617.

80. Tuleasca C, Carron R, Resseguier N, Donnet A, Roussel P, Gaudart J, Levivier M, Regis J: **Repeat Gamma Knife surgery for recurrent trigeminal neuralgia: long-term outcomes and systematic review**. *J Neurosurg* 2014, **121 Suppl**:210-221.

81. Abdi S, Datta S, Lucas LF: **Role of epidural steroids in the management of chronic spinal pain: a systematic review of effectiveness and complications**. *Pain Physician* 2005, **8**(1):127-143.

82. Carroll DG, Kline KM, Malnar KF: **Role of topiramate for the treatment of painful diabetic peripheral neuropathy**. *Pharmacotherapy:The Journal of Human Pharmacology & Drug Therapy* 2004, **24**(9):1186-1193.

83. Miller LE, Block JE: **Safety and effectiveness of bone allografts in anterior cervical discectomy and fusion surgery**. *Spine* 2011, **36**(24):2045-2050.

84. Lihua P, Su M, Zejun Z, Ke W, Bennett Michael I: **Spinal cord stimulation for cancer-related pain in adults**. In: *Cochrane Database of Systematic Reviews.* John Wiley & Sons, Ltd; 2013.

85. Turner JA, Loeser JD, Deyo RA, Sanders SB: **Spinal cord stimulation for patients with failed back surgery syndrome or complex regional pain syndrome: A systematic review of effectiveness and complications**. *Pain* 2004, **108**(1-2):137-147.

86. Taylor RS: **Spinal cord stimulation in complex regional pain syndrome and refractory neuropathic back and leg pain/failed back surgery syndrome: results of a systematic review and meta-analysis**. *Journal of Pain & Symptom Management* 2006, **31**(4S):S13-19.

87. Fabregat G, De Andres J, Villanueva-Perez VL, Asensio-Samper JM: **Subcutaneous and perineural botulinum toxin type a for neuropathic pain: a descriptive review**. *Clin J Pain* 2013, **29**(11):1006-1012.

88. Matz PG, Ryken TC, Groff MW, Vresilovic EJ, Anderson PA, Heary RF, Holly LT, Kaiser MG, Mummaneni PV, Choudhri TF *et al*: **Techniques for anterior cervical decompression for radiculopathy**. *Journal of Neurosurgery Spine* 2009, **11**(2):183-197.

89. Müller R, Peter C, Cieza A, Geyh S: **The role of social support and social skills in people with spinal cord injury-a systematic review of the literature**. *Spinal Cord* 2012, **50**(2):94-106.

90. Singh D, Kennedy DH: **The use of gabapentin for the treatment of postherpetic neuralgia**. *Clin Ther* 2003, **25**(3):852-889.

91. Ziegler D: **Thioctic acid for patients with symptomatic diabetic polyneuropathy: a critical review**. *Treatments in Endocrinology* 2004, **3**(3):173-189.

92. Padilla M, Clark GT, Merrill RL: **Topical medications for orofacial neuropathic pain: a review**. *Journal of the American Dental Association (JADA)* 2000, **131**(2):184-195.

93. Johnson MI, Bjordal JM: **Transcutaneous electrical nerve stimulation for the management of painful conditions: Focus on neuropathic pain**. *Expert Review of Neurotherapeutics* 2011, **11**(5):735-753.

94. Ziegler D, Nowak H, Kempler P, Vargha P, Low PA: **Treatment of symptomatic diabetic polyneuropathy with the antioxidant α-lipoic acid: a meta-analysis**. *Diabet Med* 2004, **21**(2):114-121.

95. Joss JD: **Tricyclic Antidepressant Use in Diabetic Neuropathy**. *Ann Pharmacother* 1999, **33**(9):996-1000.

96. Laird MA, Gidal BE: **Use of Gabapentin in the Treatment of Neuropathic Pain**. *Ann Pharmacother* 2000, **34**(6):802-807.

97. Vance CGT, Dailey DL, Rakel BA, Sluka KA: **Using TENS for pain control: the state of the evidence**. *Pain Management* 2014, **4**(3):197-209.

98. Tatli M, Satici O, Kanpolat Y, Sindou M: **Various surgical modalities for trigeminal neuralgia: literature study of respective long-term outcomes**. *Acta Neurochir (Wien)* 2008, **150**(3):243-255.

99. Andrews JC: **Vulvodynia Interventions—Systematic Review and Evidence Grading**. *Obstet Gynecol Surv* 2011, **66**(5):299-315.

100. Ahn NU, Ahn UM, Nallamshetty L, Springer BD, Buchowski JM, Funches L, Garrett ES, Kostuik JP, Kebaish KM, Sponseller PD: **Cauda equina syndrome in ankylosing spondylitis (the CES-AS syndrome): meta-analysis of outcomes after medical and surgical treatments**. *J Spinal Disord* 2001, **14**(5):427-433.

101. Cetas JS, Saedi T, Burchiel KJ: **Destructive procedures for the treatment of nonmalignant pain: a structured literature review**. *J Neurosurg* 2008, **109**(3):389-404.

102. Criner TM, Perdun CS: **Dextromethorphan and diabetic neuropathy**. *Ann Pharmacother* 1999, **33**(11):1221-1223.

103. Jimenez DF, Gibbs SR, Clapper AT: **Endoscopic treatment of carpal tunnel syndrome: a critical review**. *J Neurosurg* 1998, **88**(5):817-826.

104. Dy SM: **Evidence-based approaches to pain in advanced cancer**. *Cancer Journal* 2010, **16**(5):500-506.

105. Zakrzewska JM, Coakham HB: **Microvascular decompression for trigeminal neuralgia: update**. *Curr Opin Neurol* 2012, **25**(3):296-301.

106. Lopez BC, Hamlyn PJ, Zakrzewska JM: **Systematic review of ablative neurosurgical techniques for the treatment of trigeminal neuralgia**. *Neurosurgery* 2004, **54**(4):973-982; discussion 982-973.

107. Watson CP: **The treatment of neuropathic pain: antidepressants and opioids**. *Clin J Pain* 2000, **16**(2 Suppl):S49-55.

108. Freeman BJC, Mehdian R: **Intradiscal electrothermal therapy, percutaneous discectomy, and nucleoplasty: what is the current evidence?** *Current Pain & Headache Reports* 2008, **12**(1):14-21.

109. McCormick Z, Chien GC, Huang M, Harden RN: **A SYSTEMATIC REVIEW OF PHARMACOLOGIC TREATMENT FOR PHANTOM LIMB PAIN ORGANIZED BY PATHOPHYSIOLOGIC TARGETS**. *Am J Phys Med Rehabil* 2014:a77-a77.

110. Gangi A, Tsoumakidou G, Buy X, Cabral JF, Garnon J: **Percutaneous techniques for cervical pain of discal origin**. *Seminars in Musculoskeletal Radiology* 2011, **15**(2):172-180.

111. Choi S, Brull R: **Is ultrasound guidance advantageous for interventional pain management? A review of acute pain outcomes**. *Anesth Analg* 2011, **113**(3):596-604.

112. Alentado VJ, Lubelski D, Steinmetz MP, Benzel EC, Mroz TE: **Optimal duration of conservative management prior to surgery for cervical and lumbar radiculopathy: a literature review**. *Global spine journal* 2014, **4**(4):279-286.

113. Wood MJ, Kay R, Dworkin RH, Soong SJ, Whitley RJ: **Oral acyclovir therapy accelerates pain resolution in patients with herpes zoster: a meta-analysis of placebo-controlled trials**. *Clin Infect Dis* 1996, **22**(2):341-347.

114. Lancaster T, Silagy C, Gray S: **Primary care management of acute herpes zoster: systematic review of evidence from randomized controlled trials**. *Br J Gen Pract* 1995, **45**(390):39-45.

115. Cao H, Zhu C, Liu J: **Wet cupping therapy for treatment of herpes zoster: a systematic review of randomized controlled trials**. *Altern Ther Health Med* 2010, **16**(6):48-54.

116. Lycka BAS: **Postherpetic neuralgia and systemic corticosteroid therapy. Efficacy and safety**. *Int J Dermatol* 1990, **29**(7):523-527.

117. Furlan AD, van Tulder M, Cherkin D, Tsukayama H, Lao L, Koes B, Berman B: **Acupuncture and dry-needling for low back pain**. In: *Cochrane Database of Systematic Reviews.* John Wiley & Sons, Ltd; 2005.

118. Ammendolia C, Stuber KJ, Rok E, Rampersaud R, Kennedy CA, Pennick V, Steenstra IA, de Bruin LK, Furlan Andrea D: **Nonoperative treatment for lumbar spinal stenosis with neurogenic claudication**. In: *Cochrane Database of Systematic Reviews.* John Wiley & Sons, Ltd; 2013.

119. Machado LA, Kamper SJ, Herbert RD, Maher CG, McAuley JH: **Analgesic effects of treatments for non-specific low back pain: a meta-analysis of placebo-controlled randomized trials (Structured abstract)**. In: *Rheumatology (Oxford).* 2009: 520-527.

120. Henschke N, Ostelo RWJG, van Tulder MW, Vlaeyen JWS, Morley S, Assendelft WJJ, Main CJ: **Behavioural treatment for chronic low-back pain**. In: *Cochrane Database of Systematic Reviews.* John Wiley & Sons, Ltd; 2010.

121. Lin JH, Chiu TTW, Hu J: **Chinese manipulation for mechanical neck pain: a systematic review**. *Clin Rehabil* 2012, **26**(11):963-973.

122. Botelho RV, Dos Santos Buscariolli Y, de Barros Vasconcelos Fernandes Serra MVF, Bellini MNP, Bernardo WM: **The choice of the best surgery after single level anterior cervical spine discectomy: a systematic review**. *The open orthopaedics journal* 2012, **6**:121-128.

123. Richman JM, Liu SS, Courpas G, Wong R, Rowlingson AJ, McGready J, Cohen SR, Wu CL: **Does continuous peripheral nerve block provide superior pain control to opioids? A meta-analysis**. *Anesth Analg* 2006, **102**(1):248-257.

124. Chung JW, Zeng Y, Wong TK: **Drug therapy for the treatment of chronic nonspecific low back pain: systematic review and meta-analysis**. *Pain Physician* 2013, **16**(6):E685-704.

125. Overdevest GM, Jacobs W, Vleggeert-Lankamp C, Thomé C, Gunzburg R, Peul W: **Effectiveness of posterior decompression techniques compared with conventional laminectomy for lumbar stenosis**. In: *Cochrane Database of Systematic Reviews.* John Wiley & Sons, Ltd; 2015.

126. Zhang J, Ho KY, Wang Y: **Efficacy of pregabalin in acute postoperative pain: a meta-analysis**. *Br J Anaesth* 2011, **106**(4):454-462.

127. Geurts JW, van Wijk RM, Stolker RJ, Groen GJ: **Efficacy of radiofrequency procedures for the treatment of spinal pain: a systematic review of randomized clinical trials**. *Regional Anesthesia & Pain Medicine* 2001, **26**(5):394-400.

128. Oltean H, Robbins C, van Tulder MW, Berman BM, Bombardier C, Gagnier JJ: **Herbal medicine for low-back pain**. In: *Cochrane Database of Systematic Reviews.* John Wiley & Sons, Ltd; 2014.

129. Lunn MPT, Nobile-Orazio E: **Immunotherapy for IgM anti-myelin-associated glycoprotein paraprotein-associated peripheral neuropathies**. *Cochrane Database of Systematic Reviews* 2012, **5**:CD002827.

130. Staal BJ, de Bie RA, de Vet HCW, Hildebrandt J, Nelemans P: **Injection therapy for subacute and chronic low back pain: an updated Cochrane review**. *Spine* 2009, **34**(1):49-59.

131. Wasiak J, Mahar PD, McGuinness SK, Spinks A, Danilla S, Cleland H, Tan HB: **Intravenous lidocaine for the treatment of background or procedural burn pain**. In: *Cochrane Database of Systematic Reviews.* John Wiley & Sons, Ltd; 2014.

132. Rømsing J, Møiniche S, Ostergaard D, Dahl JB: **Local infiltration with NSAIDs for postoperative analgesia: evidence for a peripheral analgesic action**. *Acta Anaesthesiol Scand* 2000, **44**(6):672-683.

133. Yousefi-Nooraie R, Schonstein E, Heidari K, Rashidian A, Pennick V, Akbari-Kamrani M, Irani S, Shakiba B, Mortaz Hejri S, Jonaidi A-R *et al*: **Low level laser therapy for nonspecific low-back pain**. In: *Cochrane Database of Systematic Reviews.* John Wiley & Sons, Ltd; 2008.

134. Furlan AD, Imamura M, Dryden T, Irvin E: **Massage for low-back pain**. In: *Cochrane Database of Systematic Reviews.* John Wiley & Sons, Ltd; 2008.

135. Patel KC, Gross A, Graham N, Goldsmith CH, Ezzo J, Morien A, Peloso PMJ: **Massage for mechanical neck disorders**. In: *Cochrane Database of Systematic Reviews.* John Wiley & Sons, Ltd; 2012.

136. Nicolucci A, Carinci F, Cavaliere D, Scorpiglione N, Belfiglio M, Labbrozzi D, Mari E, Benedetti MM, Tognoni G, Liberati A: **A meta-analysis of trials on aldose reductase inhibitors in diabetic peripheral neuropathy. The Italian Study Group. The St. Vincent Declaration**. *Diabet Med* 1996, **13**(12):1017-1026.

137. Nicholson AB: **Methadone for cancer pain**. In: *Cochrane Database of Systematic Reviews.* John Wiley & Sons, Ltd; 2007.

138. Kamper SJ, Apeldoorn AT, Chiarotto A, Smeets RJEM, Ostelo RWJG, Guzman J, van Tulder MW: **Multidisciplinary biopsychosocial rehabilitation for chronic low back pain**. In: *Cochrane Database of Systematic Reviews.* John Wiley & Sons, Ltd; 2014.

139. Aggarwal VR, Lovell K, Peters S, Javidi H, Joughin A, Goldthorpe J: **Psychosocial interventions for the management of chronic orofacial pain**. In: *Cochrane Database of Systematic Reviews.* John Wiley & Sons, Ltd; 2011.

140. Straube S, Derry S, Moore RA, Wiffen PJ, McQuay HJ: **Single dose oral gabapentin for established acute postoperative pain in adults**. In: *Cochrane Database of Systematic Reviews.* John Wiley & Sons, Ltd; 2010.

141. Moore RA, Derry S, Mason L, McQuay HJ, Edwards J: **Single dose oral indometacin for the treatment of acute postoperative pain**. In: *Cochrane Database of Systematic Reviews.* John Wiley & Sons, Ltd; 2004.

142. Toms L, McQuay HJ, Derry S, Moore RA: **Single dose oral paracetamol (acetaminophen) for postoperative pain in adults**. In: *Cochrane Database of Systematic Reviews.* John Wiley & Sons, Ltd; 2008.

143. Toms L, Derry S, Moore RA, McQuay HJ: **Single dose oral paracetamol (acetaminophen) with codeine for postoperative pain in adults**. In: *Cochrane Database of Systematic Reviews.* John Wiley & Sons, Ltd; 2009.

144. Bulley S, Derry S, Moore RA, McQuay HJ: **Single dose oral rofecoxib for acute postoperative pain in adults**. In: *Cochrane Database of Systematic Reviews.* John Wiley & Sons, Ltd; 2009.

145. Pagnini F, Bosma CM, Phillips D, Langer E: **Symptom changes in multiple sclerosis following psychological interventions: A systematic review**. *BMC Neurology* 2014, **14**.

146. Nnoaham KE, Kumbang J: **Transcutaneous electrical nerve stimulation (TENS) for chronic pain**. In: *Cochrane Database of Systematic Reviews.* John Wiley & Sons, Ltd; 2014.

147. Khadilkar A, Odebiyi DO, Brosseau L, Wells GA: **Transcutaneous electrical nerve stimulation (TENS) versus placebo for chronic low-back pain**. *Cochrane Database Syst Rev* 2008(4):CD003008.

148. Hadley G, Derry S, Moore RA, Wiffen PJ: **Transdermal fentanyl for cancer pain**. In: *Cochrane Database of Systematic Reviews.* John Wiley & Sons, Ltd; 2013.

149. Stork ACJ, Lunn MPT, Niermeijer J, Nobile-Orazio E: **Treatment for IgG and IgA paraproteinaemic neuropathy**. *Cochrane Database of Systematic Reviews* 2007(1):CD005376.

150. Giometto B, Vitaliani R, Lindeck-Pozza E, Grisold W, Vedeler C: **Treatment for paraneoplastic neuropathies**. *Cochrane Database of Systematic Reviews* 2012, **12**:CD007625.

151. Montano N, Papacci F, Cioni B, Di Bonaventura R, Meglio M: **What is the best treatment of drug-resistant trigeminal neuralgia in patients affected by multiple sclerosis? A literature analysis of surgical procedures**. *Clin Neurol Neurosurg* 2013, **115**(5):567-572.

152. Carstensen M, Moller AM: **Adding ketamine to morphine for intravenous patient-controlled analgesia for acute postoperative pain: a qualitative review of randomized trials**. *Br J Anaesth* 2010, **104**(4):401-406.

153. Mark DH, Aronson N, Ziegler KM, Bonnell CJ, Hines KL: **Low-level laser therapy for carpal tunnel syndrome and chronic neck pain**. In: *Database of Abstracts of Reviews of Effects.* Blue Cross and Blue Shield Association, Technology Evaluation Center; 2010: 1.

154. Macaulay J, Cameron M, Vaughan B: **The effectiveness of manual therapy for neck pain: a systematic review of the literature**. *Physical Therapy Reviews* 2007, **12**(3):261-267.

155. Paley CA, Johnson MI, Tashani OA, Bagnall A-M: **Acupuncture for cancer pain in adults**. In: *Cochrane Database of Systematic Reviews.* John Wiley & Sons, Ltd; 2011.

156. Saarto T, Wiffen PJ: **Antidepressants for neuropathic pain: A Cochrane review**. *J Neurol Neurosurg Psychiatry* 2010, **81**(12):1372-1373.

157. Straube S, Derry S, Moore RA, Cole P: **Cervico-thoracic or lumbar sympathectomy for neuropathic pain and complex regional pain syndrome**. In: *Cochrane Database of Systematic Reviews.* John Wiley & Sons, Ltd; 2013.

158. Chaparro LE, Wiffen PJ, Moore RA, Gilron I: **Combination pharmacotherapy for the treatment of neuropathic pain in adults**. *Cochrane Database of Systematic Reviews* 2012(7).

159. Eisenberg E, McNicol ED, Carr DB: **Efficacy and safety of opioid agonists in the treatment of neuropathic pain of nonmalignant origin: systematic review and meta-analysis of randomized controlled trials**. *JAMA* 2005, **293**(24):3043-3052.

160. Koes BW, Scholten RJ, Mens JM, Bouter LM: **Efficacy of epidural steroid injections for low-back pain and sciatica: a systematic review of randomized clinical trials (Structured abstract)**. In: *Pain.* 1995: 279-288.

161. Eisenberg E, McNicol ED, Carr DB: **Efficacy of mu-opioid agonists in the treatment of evoked neuropathic pain: Systematic review of randomized controlled trials**. *Eur J Pain* 2006, **10**(8):667-676.

162. Bell RF, Eccleston C, Kalso EA: **Ketamine as an adjuvant to opioids for cancer pain**. In: *Cochrane Database of Systematic Reviews.* John Wiley & Sons, Ltd; 2012.

163. Stanton TR, Wand BM, Carr DB, Birklein F, Wasner GL, O'Connell NE: **Local anaesthetic sympathetic blockade for complex regional pain syndrome**. *Cochrane Database of Systematic Reviews* 2013(8).

164. Xing D, Ma X-L, Ma J-X, Wang J, Ma T, Chen Y: **A meta-analysis of cervical arthroplasty compared to anterior cervical discectomy and fusion for single-level cervical disc disease**. *Journal of Clinical Neuroscience* 2013, **20**(7):970-978.

165. Salt E, Wright C, Kelly S, Dean A: **A systematic literature review on the effectiveness of non-invasive therapy for cervicobrachial pain**. *Manual Therapy* 2011, **16**(1):53-65.

166. McQuay HJ, Tramer M, Nye BA, Carroll D, Wiffen PJ, Moore RA: **A systematic review of antidepressants in neuropathic pain**. *Pain* 1996, **68**(2-3):217-227.

167. Collins S, Sigtermans MJ, Dahan A, Zuurmond WWA, Perez RSGM: **NMDA receptor antagonists for the treatment of neuropathic pain**. *Pain Medicine* 2010, **11**(11):1726-1742.

168. McNicol ED, Midbari A, Eisenberg E: **Opioids for neuropathic pain**. *Cochrane Database of Systematic Reviews* 2013(8).

169. Leininger B, Bronfort G, Evans R, Reiter T: **Spinal manipulation or mobilization for radiculopathy: a systematic review**. *Phys Med Rehabil Clin N Am* 2011, **22**(1):105-125.

170. Nikolaidis I, Fouyas IP, Sandercock PAG, Statham PF: **Surgery for cervical radiculopathy or myelopathy**. In: *Cochrane Database of Systematic Reviews.* John Wiley & Sons, Ltd; 2010.

171. Tremont-Lukats IW, Challapalli V, McNicol ED, Lau J, Carr DB: **Systemic administration of local anesthetics to relieve neuropathic pain: a systematic review and meta-analysis**. *Anesth Analg* 2005, **101**(6):1738-1749.

172. Duehmke RM, Hollingshead J, Cornblath DR: **Tramadol for neuropathic pain**. In: *Cochrane Database of Systematic Reviews.* John Wiley & Sons, Ltd; 2006.

173. Ang CD, Alviar MJM, Dans AL, Bautista-Velez GGP, Villaruz-Sulit MVC, Tan JJ, Co HU, Bautista MRM, Roxas AA: **Vitamin B for treating peripheral neuropathy**. In: *Cochrane Database of Systematic Reviews.* John Wiley & Sons, Ltd; 2008.

174. Chu SH, Lee YJ, Lee ES, Geng Y, Wang XS, Cleeland CS: **Current use of drugs affecting the central nervous system for chemotherapy-induced peripheral neuropathy in cancer patients: a systematic review**. *Support Care Cancer* 2015, **23**(2):513-524.

175. Dahm KT, Brurberg KG, Jamtvedt G, Hagen KB: **Advice to rest in bed versus advice to stay active for acute low-back pain and sciatica**. In: *Cochrane Database of Systematic Reviews.* John Wiley & Sons, Ltd; 2010.

176. Finnerup NB, Otto M, McQuay HJ, Jensen TS, Sindrup SH: **Algorithm for neuropathic pain treatment: an evidence based proposal**. *Pain* 2005, **118**(3):289-305.

177. Vargas-Espinosa M-L, Sanmarti-Garcia G, Vazquez-Delgado E, Gay-Escoda C: **Antiepileptic drugs for the treatment of neuropathic pain: a systematic review**. *Medicina Oral, Patologia Oral y Cirugia Bucal* 2012, **17**(5):e786-793.

178. Romano CL, Romano D, Lacerenza M: **Antineuropathic and antinociceptive drugs combination in patients with chronic low back pain: a systematic review**. *Pain research and treatment* 2012, **2012**:154781.

179. Boselie TFM, Willems PC, van Mameren H, de Bie R, Benzel EC, van Santbrink H: **Arthroplasty versus fusion in single-level cervical degenerative disc disease**. In: *Cochrane Database of Systematic Reviews.* John Wiley & Sons, Ltd; 2012.

180. Waseem Z, Boulias C, Gordon A, Ismail F, Sheean G, Furlan Andrea D: **Botulinum toxin injections for low-back pain and sciatica**. In: *Cochrane Database of Systematic Reviews.* John Wiley & Sons, Ltd; 2011.

181. Trinh K, Cui X, Wang Y-J: **Chinese herbal medicine for chronic neck pain due to cervical degenerative disc disease**. *Spine* 2010, **35**(24):2121-2127.

182. Kumar A, Maitra S, Khanna P, Baidya DK: **Clonidine for management of chronic pain: A brief review of the current evidences**. *Saudi journal of anaesthesia* 2014, **8**(1):92-96.

183. Walker BF, French SD, Grant W, Green S: **Combined chiropractic interventions for low-back pain**. In: *Cochrane Database of Systematic Reviews.* John Wiley & Sons, Ltd; 2010.

184. Van Veen NHJ, Nicholls PG, Smith WCS, Richardus JH: **Corticosteroids for treating nerve damage in leprosy**. In: *Cochrane Database of Systematic Reviews.* John Wiley & Sons, Ltd; 2007.

185. Van Veen NHJ, Schreuders TAR, Theuvenet WJ, Agrawal A, Richardus JH: **Decompressive surgery for treating nerve damage in leprosy**. In: *Cochrane Database of Systematic Reviews.* John Wiley & Sons, Ltd; 2012.

186. Sesti F, Capozzolo T, Pietropolli A, Collalti M, Bollea MR, Piccione E: **Dietary therapy: a new strategy for management of chronic pelvic pain**. *Nutrition research reviews* 2011, **24**(1):31-38.

187. Gadgil N, Viswanathan A: **DREZotomy in the treatment of cancer pain: a review**. *Stereotact Funct Neurosurg* 2012, **90**(6):356-360.

188. Brettschneider J, Kurent J, Ludolph A: **Drug therapy for pain in amyotrophic lateral sclerosis or motor neuron disease**. In: *Cochrane Database of Systematic Reviews.* John Wiley & Sons, Ltd; 2013.

189. Sultan A, Gaskell H, Derry S, Moore RA: **Duloxetine for painful diabetic neuropathy and fibromyalgia pain: Systematic review of randomised trials**. *BMC Neurology* 2008, **8**.

190. Lunn MPT, Hughes RAC, Wiffen PJ: **Duloxetine for treating painful neuropathy, chronic pain or fibromyalgia**. In: *Cochrane Database of Systematic Reviews.* John Wiley & Sons, Ltd; 2014.

191. Hilde G, Bo K: **Effect of exercise in the treatment of chronic low back pain: a systematic review, emphasising type and dose of exercise (Structured abstract)**. In: *Physical Therapy Reviews.* 1998: 107-117.

192. Thrane S: **Effectiveness of integrative modalities for pain and anxiety in children and adolescents with cancer: a systematic review**. *J Pediatr Oncol Nurs* 2013, **30**(6):320-332.

193. Zhang WY, Li Wan Po A: **The effectiveness of topically applied capsaicin. A meta-analysis**. *Eur J Clin Pharmacol* 1994, **46**(6):517-522.

194. Kroeling P, Gross A, Graham N, Burnie S, J., Szeto G, Goldsmith CH, Haines T, Forget M: **Electrotherapy for neck pain**. In: *Cochrane Database of Systematic Reviews.* John Wiley & Sons, Ltd; 2013.

195. Choi HJ, Hahn S, Kim CH, Jang BH, Park S, Lee SM, Park J-Y, Chung CK, Park B-J: **Epidural steroid injection therapy for low back pain: a meta-analysis**. *Int J Technol Assess Health Care* 2013, **29**(3):244-253.

196. Carr DB, Goudas LC, Balk EM, Bloch R, Ioannidis JPA, Lau J: **Evidence report on the treatment of pain in cancer patients**. *J Natl Cancer Inst* 2004, **Monographs.**(32):23-31.

197. Blum K, Chen ALC, Chen TJH, Prihoda TJ, Schoolfield J, DiNubile N, Waite RL, Arcuri V, Kerner M, Braverman ER *et al*: **The H-Wave device is an effective and safe non-pharmacological analgesic for chronic pain: a meta-analysis**. *Adv Ther* 2008, **25**(7):644-657.

198. Devulder J, Jacobs A, Richarz U, Wiggett H: **Impact of opioid rescue medication for breakthrough pain on the efficacy and tolerability of long-acting opioids in patients with chronic non-malignant pain (Structured abstract)**. In: *Br J Anaesth.* 2009: 576-585.

199. Engers AJ, Jellema P, Wensing M, van der Windt DA, Grol R, van Tulder MW: **Individual patient education for low back pain**. *Cochrane Database of Systematic Reviews* 2008(1):CD004057.

200. Noble M, Treadwell JR, Tregear SJ, Coates VH, Wiffen PJ, Akafomo C, Schoelles KM: **Long-term opioid management for chronic noncancer pain**. *Cochrane Database Syst Rev* 2010(1):CD006605.

201. Gross A, Miller J, D'Sylva J, Burnie SJ, Goldsmith CH, Graham N, Haines T, Brønfort G, Hoving JL: **Manipulation or Mobilisation for Neck Pain**. In: *Cochrane Database of Systematic Reviews.* John Wiley & Sons, Ltd; 2010.

202. Iskedjian M, Bereza B, Gordon A, Piwko C, Einarson TR: **Meta-analysis of cannabis based treatments for neuropathic and multiple sclerosis-related pain**. *Curr Med Res Opin* 2007, **23**(1):17-24.

203. Lee Y-C, Chen P-P: **A review of SSRIs and SNRIs in neuropathic pain**. *Expert opinion on pharmacotherapy* 2010, **11**(17):2813-2825.

204. Humble SR, Dalton AJ, Li L: **A systematic review of therapeutic interventions to reduce acute and chronic post‐surgical pain after amputation, thoracotomy or mastectomy**. *European Journal of Pain* 2014.

205. Haroutiunian S, McNicol ED, Lipman AG: **Methadone for chronic non-cancer pain in adults**. In: *Cochrane Database of Systematic Reviews.* John Wiley & Sons, Ltd; 2012.

206. Derry S, Gill D, Phillips T, Moore RA: **Milnacipran for neuropathic pain and fibromyalgia in adults**. In: *Cochrane Database of Systematic Reviews.* John Wiley & Sons, Ltd; 2012.

207. O'Connell NE, Wand BM, Marston L, Spencer S, DeSouza LH: **Non-invasive brain stimulation techniques for chronic pain**. In: *Cochrane Database of Systematic Reviews.* John Wiley & Sons, Ltd; 2014.

208. Boldt I, Eriks-Hoogland I, Brinkhof MWG, de Bie R, Joggi D, von Elm E: **Non-pharmacological interventions for chronic pain in people with spinal cord injury**. In: *Cochrane Database of Systematic Reviews.* John Wiley & Sons, Ltd; 2014.

209. Roelofs PD, Deyo RA, Koes BW, Scholten RJ, van Tulder MW: **Non-steroidal anti-inflammatory drugs for low back pain**. *Cochrane Database Syst Rev* 2008(1):CD000396.

210. McNicol ED, Strassels S, Goudas L, Lau J, Carr DB: **NSAIDS or paracetamol, alone or combined with opioids, for cancer pain**. In: *Cochrane Database of Systematic Reviews.* John Wiley & Sons, Ltd; 2005.

211. Chaparro LE, Furlan AD, Deshpande A, Mailis-Gagnon A, Atlas S, Turk DC: **Opioids compared to placebo or other treatments for chronic low-back pain**. In: *Cochrane Database of Systematic Reviews.* John Wiley & Sons, Ltd; 2013.

212. Deyo RA, Von Korff M, Duhrkoop D: **Opioids for low back pain**. *BMJ* 2015, **350**:g6380.

213. Zeppetella G, Davies Andrew N: **Opioids for the management of breakthrough pain in cancer patients**. In: *Cochrane Database of Systematic Reviews.* John Wiley & Sons, Ltd; 2013.

214. Kalso E, Edwards JE, Moore RA, McQuay HJ: **Opioids in chronic non-cancer pain: systematic review of efficacy and safety**. *Pain* 2004, **112**(3):372-380.

215. Welsch P, Sommer C, Schiltenwolf M, Hauser W: **[Opioids in chronic noncancer pain-are opioids superior to nonopioid analgesics? : A systematic review and meta-analysis of efficacy, tolerability and safety in randomized head-to-head comparisons of opioids versus nonopioid analgesics of at least four week's duration]**. *Der Schmerz* 2015, **29**(1):85-95.

216. Wiffen PJ, Wee B, Moore RA: **Oral morphine for cancer pain**. In: *Cochrane Database of Systematic Reviews.* John Wiley & Sons, Ltd; 2013.

217. van Tulder MW, Koes B, Seitsalo S, Malmivaara A: **Outcome of invasive treatment modalities on back pain and sciatica: an evidence-based review**. *Eur Spine J* 2006, **15 Suppl 1**:S82-92.

218. Schmidt-Hansen M, Bennett MI, Arnold S, Bromham N, Hilgart JS: **Oxycodone for cancer-related pain**. In: *Cochrane Database of Systematic Reviews.* John Wiley & Sons, Ltd; 2015.

219. Gaskell H, Moore RA, Derry S, Stannard C: **Oxycodone for neuropathic pain and fibromyalgia in adults**. In: *Cochrane Database of Systematic Reviews.* John Wiley & Sons, Ltd; 2014.

220. Liu J, Wang L-N, McNicol Ewan D: **Pharmacological treatment for pain in Guillain-Barré syndrome**. In: *Cochrane Database of Systematic Reviews.* John Wiley & Sons, Ltd; 2013.

221. Moore RA, Straube S, Wiffen PJ, Derry S, McQuay HJ: **Pregabalin for acute and chronic pain in adults**. In: *Cochrane Database of Systematic Reviews.* John Wiley & Sons, Ltd; 2009.

222. Baidya DK, Agarwal A, Khanna P, Arora MK: **Pregabalin in acute and chronic pain**. *Journal of Anaesthesiology Clinical Pharmacology* 2011, **27**(3):307-314.

223. Vossen H, Monsieur D, van Os J, Leue C: **[Serotonin-noradrenalin reuptake inhibitors in the treatment of non-malignant pain syndromes; a systematic review]**. *Tijdschrift voor Psychiatrie* 2009, **51**(11):831-840.

224. Jacobs WC, Anderson PG, Limbeek J, Willems PC, Pavlov P: **Single or double-level anterior interbody fusion techniques for cervical degenerative disc disease**. *Cochrane Database Syst Rev* 2004(4):CD004958.

225. Rubinstein SM, Terwee CB, Assendelft WJJ, de Boer MR, van Tulder MW: **Spinal manipulative therapy for acute low-back pain**. In: *Cochrane Database of Systematic Reviews.* John Wiley & Sons, Ltd; 2012.

226. Rubinstein SM, van Middelkoop M, Assendelft WJJ, de Boer MR, van Tulder MW: **Spinal manipulative therapy for chronic low-back pain**. In: *Cochrane Database of Systematic Reviews.* John Wiley & Sons, Ltd; 2011.

227. Benyamin R, Singh V, Parr AT, Conn A, Diwan S, Abdi S: **Systematic review of the effectiveness of cervical epidurals in the management of chronic neck pain (Structured abstract)**. In: *Pain Physician.* 2009: 137-157.

228. Martell BA, O'Connor PG, Kerns RD, Becker WC, Morales KH, Kosten TR, Fiellin DA: **Systematic review: opioid treatment for chronic back pain: prevalence, efficacy, and association with addiction (Structured abstract)**. In: *Ann Intern Med.* 2007: 116-127.

229. Granot R, Day RO, Cohen ML, Murnion B, Garrick R: **Targeted pharmacotherapy of evoked phenomena in neuropathic pain: a review of the current evidence**. *Pain Med* 2007, **8**(1):48-64.

230. Ebadi S, Henschke N, Nakhostin AN, Fallah E, van Tulder MW: **Therapeutic ultrasound for chronic low-back pain**. In: *Cochrane Database of Systematic Reviews.* John Wiley & Sons, Ltd; 2014.

231. Jacobs W, Van der Gaag NA, Tuschel A, de Kleuver M, Peul W, Verbout AJ, Oner FC: **Total disc replacement for chronic back pain in the presence of disc degeneration**. In: *Cochrane Database of Systematic Reviews.* John Wiley & Sons, Ltd; 2012.

232. Cossins L, Okell RW, Cameron H, Simpson B, Poole HM, Goebel A: **Treatment of complex regional pain syndrome in adults: A systematic review of randomized controlled trials published from June 2000 to February 2012**. *European Journal of Pain* 2013, **17**(2):158-173.

233. Tengrungsun T, Mitriattanakul S, Buranaprasertsuk P, Suddhasthir T: **Is low level laser effective for the treatment of orofacial pain?: A systematic review**. *Cranio* 2012, **30**(4):280-285.

234. Rabinovitch DL, Peliowski A, Furlan AD: **Influence of lumbar epidural injection volume on pain relief for radicular leg pain and/or low back pain**. *Spine Journal: Official Journal of the North American Spine Society* 2009, **9**(6):509-517.

235. Lee MS, Pittler MH, Ernst E: **Is reiki beneficial for pain management? (Structured abstract)**. In: *Focus on Alternative and Complementary Therapies.* 2008: 78-81.

236. Morales-Osorio MA, Mejía-Mejía JM: **Treatment with graded motor imagery in phantom member syndrome with pain: A systematic review [Spanish]**. *Rehabilitacion* 2012, **46**(4):310-316.

237. White CM, Pritchard J, Turner-Stokes L: **Exercise for people with peripheral neuropathy**. In: *Cochrane Database of Systematic Reviews.* John Wiley & Sons, Ltd; 2004.

238. Chen W, Yang G-Y, Liu B, Manheimer E, Liu J-P: **Manual acupuncture for treatment of diabetic peripheral neuropathy: a systematic review of randomized controlled trials**. *PLoS ONE [Electronic Resource]* 2013, **8**(9):e73764.

239. Han T, Bai J, Liu W, Hu Y: **A systematic review and meta-analysis of alpha-lipoic acid in the treatment of diabetic peripheral neuropathy**. *European Journal of Endocrinology* 2012, **167**(4):465-471.

240. Wu J, Zhang X, Zhang B: **Efficacy and safety of puerarin injection in treatment of diabetic peripheral neuropathy: a systematic review and meta-analysis of randomized controlled trials**. *J Tradit Chin Med* 2014, **34**(4):401-410.

241. Coulthard P, Kushnerev E, Yates JM, Walsh T, Patel N, Bailey E, Renton TF: **Interventions for iatrogenic inferior alveolar and lingual nerve injury**. In: *Cochrane Database of Systematic Reviews.* John Wiley & Sons, Ltd; 2014.

242. Chin YH, Lim KH, Poh BK, Koh D: **Carpal tunnel syndrome: splinting or surgery? A systematic review (Provisional abstract)**. In: *Singapore General Hospital Proceedings.* 2005: 128-132.

243. Huntley AL, Ernst E: **CAM for painful diabetic neuropathy: a systematic review... 12th Annual Symposium on Complementary Health Care -- Abstracts: 19th-21st September 2005, Exeter, UK**. *Focus on Alternative & Complementary Therapies* 2005, **10**:26-26.

244. Yang LJ, Lin P, Liang JH, Zhen JC: **Effects and safety of pregabalin for the treatment of diabetic peripheral neuropathic pain: meta-analysis of randomized controlled trials (Provisional abstract)**. In: *Database of Abstracts of Reviews of Effects.* 2013: 1881-1885.

245. Gibson JNA, Waddell G: **Surgical interventions for lumbar disc prolapse**. In: *Cochrane Database of Systematic Reviews.* John Wiley & Sons, Ltd; 2007.
